# Global, regional, and national burden of diabetes mellitus due to metabolic factors in young adults, 1990 to 2021 and predictions to 2040: An analysis of the Global Burden of Disease Study 2021

**DOI:** 10.1097/MD.0000000000046261

**Published:** 2025-12-12

**Authors:** Guokun Sun, Yating Wen, Xiaojing Han, Qiao Wang, Yunli Wang, Yi Mao, Xiaolu Sun, Yan Zhai, Yaru Wen, Xiaoqing Han

**Affiliations:** a Department of Intensive Care Medicine, Weifang People’s Hospital, Weifang, Shandong, China; b Department of Spine Degeneration and Oncology, Weifang People’s Hospital, Weifang, Shandong, China; c Obstetrics Medical Center, Weifang People’s Hospital, Weifang, Shandong, China; d Department of General Surgery, The 80th Group Army Hospital of the Chinese People’s Liberation Army (PLA), Weifang, Shandong, China; e Health Management Center, Weifang People’s Hospital, Weifang, Shandong, China; f Department of Clinical Medicine, Shandong Second Medical University, Weifang, Shandong, China.

**Keywords:** diabetes mellitus, Global Disease Burden, metabolic factors, Young adults

## Abstract

Diabetes mellitus due to metabolic factors is a significant global public health burden, showing an increasing trend among young adults aged 24 to 49 years. This group, who are at the peak of their career development and family obligations, has received limited research attention despite its critical role in societal development. This study aims to evaluate the global, regional, and national burden of diabetes mellitus due to metabolic factors in young adults from 1990 to 2021 and project trends to 2040, using data from the Global Burden of Disease Study 2021, to inform targeted prevention and management strategies. Data for this study were obtained from the Global Burden of Disease (GBD) database spanning 1990 to 2021. The analysis focused on trends in mortality and disability-adjusted life years (DALYs), and the estimated annual percentage changes (EAPCs) in disease burden were calculated over the study period. Future trends in disease burden up to 2040 were projected using the Bayesian age-period-cohort (BAPC) model. Frontier analysis was conducted to explore potential improvement opportunities and disparities across countries categorized by development level. The analyses were stratified by gender, 21 GBD regions, 204 nations/territories, and 5 SDI quintiles. Statistical analyses and plot illustrations were conducted using the R statistical package (version 4.4.2). The global burden of diabetes mellitus due to metabolic factors exhibits significant regional variations, which are closely linked to sociodemographic index (SDI) levels. The age-standardized mortality rate (ASMR) declined, while the age-standardized disability rate (ASDR) increased from 1990 to 2021. The BAPC model predicts an increasing burden of mortality and DALYs over the next 2 decades. Meanwhile, frontier analysis reveals substantial room for improvement across all levels of development. Management of metabolic factors remains a major challenge for young people with diabetes mellitus, and targeted clinical guidelines are needed.

## 1. Introduction

Diabetes mellitus (DM) is a major global health issue, which places a significant burden on both public health systems and socioeconomic development.^[1]^ While the incidence of diabetes has declined in some countries, its prevalence has increased in most developed and developing nations in recent years.^[2]^ According to the International Diabetes Federation (IDF), an estimated 451 million adults worldwide had diabetes in 2017, with projections indicating this number could rise to 693 million by 2045 without effective preventive strategies.^[3]^ A significant contributor to this burden is the presence of metabolic risk factors, including elevated fasting plasma glucose, high body mass index (BMI), and hyperlipidemia, which are recognized as critical determinants in the development and progression of diabetes.^[4,5]^

Diabetes has historically been perceived as a disease predominantly affecting older adults. However, recent evidence indicates an alarming increase in diabetes prevalence among younger adults, particularly those aged 25 to 49 years, who frequently present with comorbid metabolic risk factors.^[6]^ Studies have reported that this age group has the highest increase in diabetes incidence, which has significant implications for healthcare systems.^[7]^ Young adults with diabetes face a prolonged disease course, an increased risk of severe complications, and a significantly higher mortality rate compared to their healthy peers.^[8]^ This population consists of individuals in their most economically and socially productive years, and the progression of diabetes during this period can have far reaching consequences for both individual health and broader societal productivity.^[9]^

In the context of an aging population, the health of younger adults becomes increasingly critical, as any decline in their workforce participation exacerbates the strain on healthcare systems and negatively impacts national productivity.^[10]^ Thus, the issue of metabolic risk factor-induced diabetes in young adults is not only a pressing health concern but also a potential threat to economic and social sustainability.^[11]^ However, there is limited evidence regarding the burden of diabetes due to metabolic factors among young people globally. These gaps in knowledge may hinder policymakers in developing effective strategies at both regional and national levels to alleviate the burden of diabetes due to metabolic factors among young population.

To address these challenges, this study analyzes data from the Global Burden of Disease Study 2021 to determine the epidemiological trends in age-standardized disability-adjusted life years (DALY) and mortality rates of diabetes due to metabolic factors in young adults aged 25 to 49 years across 204 countries and territories from 1990 to 2021. The analysis is stratified by age, sex, region, country, and sociodemographic index (SDI). Understanding the epidemiological patterns and the burden of diabetes attributed to metabolic factors in young adults is crucial for informing targeted prevention strategies, early detection programs, and effective resource allocation.

## 2. Methods

### 2.1. Data acquisition

The retrospective study collected disease burden-related data using a method similar to the previous publication that systematically researched the burden of DM due to metabolic factors on a global, regional, and national scale. In the Global Burden of Disease Study 2021 (GBD 2021) Data Resources, we obtained comprehensive GBD results data using a query tool. This included estimated mortality and disability-adjusted life-years (DALYs), and risk factors for 371 diseases and injuries, for both sexes, and across 204 countries and territories.

### 2.2. Projection analysis

The Bayesian age-period-cohort (BAPC) model was applied to forecast the DALYs and mortality associated with DM due to metabolic factors for the period 2022 to 2040. In essence, this logarithmic linear Poisson framework assumes that age, period, and cohort effects exert a multiplicative influence, with each factor conforming to a Poisson distribution and utilizing a model-specific link function. Utilizing data from 2021, projections were generated based on historical trends. The 2021 data for both the number and age standardized rate linked to DM due to metabolic factors served as the baseline reference. A 1% annual increase was considered a negative scenario, while a 1% annual decline represented an optimistic projection. The BAPC model was executed using the BAPC package in R.

### 2.3. Frontier analysis

To examine the link between diabetes burden due to metabolic factors and sociodemographic development levels, we used frontier analysis to develop a model based on the age-standardized DALYs (ASDR) and mortality rates (ASMR) using SDI. Unlike traditional regression models, frontier analysis was applied to capture the nonlinear relationship between SDI and disease burden, addressing the complex factors influencing the burden. This approach identifies the lowest possible ASDR/ASMR value each country or territory could achieve based on its development, providing a benchmark for optimal performance. The gap between current and potential minimum burden highlights areas for improvement. We employed locally weighted regression (LOESS) combined with local polynomial regression with varying smoothing spans (0.3, 0.4, 0.5) to generate smooth frontier lines, revealing the nonlinear relationship between SDI and ASDR/ASMR. To ensure robustness, 1000 bootstrap samples were analyzed to calculate average ASDR and ASMR for each SDI value. The absolute difference between each country’s or territory’s 2021 ASDR/ASMR and the frontier line was measured to assess improvement potential.

### 2.4. Statistical Analysis

Numbers of mortality and DALYs, and their corresponding rates were the main indicators used to describe the burden of DM due to metabolic factors. Each rate is reported per 100 000 population, along with 95% uncertainty interval according to the GBD algorithm. The dynamics of DM due to metabolic factors were analyzed by calculating EAPCs to identify temporal trends in the disease burden; 95% CIs of EAPCs were determined by linear modeling. If the upper limit of both EAPC and its 95% CI is negative, its corresponding rate shows a decreasing trend; conversely, if the lower limit of both an EAPC and its 95% CI is positive, its corresponding rate shows an increasing trend. All calculations were performed using R Studio, version 4.1.2 (R Project for Statistical Computing). All *P* values were 2-sided, and *P* < .05 was considered statistically significant.

## 3. Results

### 3.1. Global level

In 2021, there were 111,943 mortality cases of DM due to metabolic factors, with a 95% UI ranging from 103,690 to 120,764, representing a significant increase of 86.81% compared to the year 1990 (Table 1). The ASMR of DM due to metabolic factors in 2021 was recorded at a lower value of 4.02/100,000 (95% UI = 3.73–4.34) as opposed to the rate in the year 1990 which stood at 3.83/100,000 (95% UI = 3.63–4.08). Furthermore, the EAPC indicated that ASMR experienced a decline during the period between 1990 and 2021 [−0.04 (95% UI = −0.125 to 0.046)]. In 2021, the highest ASMR was observed in the 45 to 49 age group (10.40/1000, 95% UI = 9.64–11.23). The fastest growing age group is 25 to 29 years old (EAPC = 0.039, 95% CI = −0.03 to 0.109). There was a higher ASMR in males compared to females in all age group (Table S1, Supplemental Digital Content, https://links.lww.com/MD/Q795).

**Table 1:** Mortality and DALY rates of diabetes mellitus due to metabolic factors in young adults (aged 25–49 yr) by location group in 1990 and 2021, and estimated annual percentage changes from 1990 to 2021.

Location	Num_1990 (95% UI)	ASR_1990 per 100,000 persons (95% UI)	Num_2021 (95% UI)	ASR_2021 per 100,000 persons (95% UI)	EAPC (95% CI)	Num_1990 (95% UI)	ASR_1990 per 100,000 persons (95% UI)	Num_2021 (95% UI)	ASR_2021 per 100,000 persons (95% UI)	EAPC (95% CI)
Global	59,923 (56,816–63,775)	3.83 (3.63–4.08)	111,943 (103,690–120,764)	4.02 (3.73–4.34)	−0.04 (−0.125 to 0.046)	5,866,826 (4,849,989-–7,114,242)	367.76 (304.68–444.97)	15,754,105 (12,318,727-–20,033,528)	569.45 (444.94–724.43)	1.308 (1.246 to 1.369)
SDI										
High-SDI	8592 (8374–8829)	2.65 (2.59–2.73)	8889 (8418–9476)	2.19 (2.07–2.33)	−0.901 (−1.076 to −0.725)	922,532 (748,927–1,130,861)	284.15 (230.64–348.29)	1,932,068 (1,419,475–2,557,523)	483.12 (354.53–640.61)	1.599 (1.509 to 1.688)
High-middle SDI	7597 (7085–8174)	2.22 (2.07–2.39)	9247 (8425–10,171)	1.77 (1.61–1.94)	−1.253 (−1.441 to −1.065)	974,744 (761,958–1,246,444)	278.75 (218.27–355.55)	2,273,128 (1,636,084–3,063,193)	446.46 (320.34–602.98)	1.371 (1.254 to 1.489)
Middle SDI	20,186 (19,037–21,387)	4.15 (3.92–4.4)	40,508 (37,538–43,437)	4.34 (4.02–4.66)	−0.091 (−0.21 to 0.027)	2,038,441 (1,670,241–2,489,398)	408.06 (335.35–496.68)	5,440,677 (4,263,807–6,899,901)	590.38 (461.91–749.41)	1.051 (0.971 to 1.132)
Low-middle SDI	15,408 (13,964–17,095)	5.19 (4.7–5.75)	35,370 (31,641–39,465)	5.66 (5.06–6.31)	0.264 (0.227 to 0.302)	1,330,482 (1,123,997–1,576,255)	439.02 (371.56–519.12)	4,254,629 (3,380,242–5,347,951)	671.97 (534.64–843.69)	1.325 (1.283 to 1.366)
Low-SDI	8044 (7028–9215)	7.12 (6.22–8.15)	17,781 (15,397–20,709)	6.29 (5.45–7.33)	−0.623 (−0.7 to −0.546)	592,689 (511,219–690,586)	511.41 (441.78–594.82)	1,836,932 (1,499,522–2,292,806)	633.32 (518.27–789.34)	0.521 (0.456 to 0.585)
GBD regions										
Andean Latin America	379 (332–436)	3.78 (3.27–4.38)	890 (703–1121)	25.18 (19.69–32.27)	−0.199 (−0.373 to −0.025)	30,135 (24,941–36,564)	306.75 (254.02–371.76)	98,028 (77,526–124,849)	427.34 (338.14–544.28)	0.937 (0.861 to 1.014)
Australasia	115 (106–126)	1.49 (1.37–1.63)	141 (127–157)	1.43 (1.18–1.71)	−1.006 (−1.256 to −0.755)	13,169 (10,566–16,493)	176.6 (141.8–221.14)	26,901 (19,844–35,899)	242.15 (178.66–323.06)	0.969 (0.903 to 1.036)
Caribbean	931 (830–1058)	9.02 (8.07–10.26)	1435 (1143–1788)	6.36 (5.51–7.3)	−0.34 (−0.553 to −0.127)	78,680 (66,609–94,850)	759.04 (642.63–915.3)	175,021 (135,974–225,664)	1060.15 (823.62–1366.63)	0.964 (0.845 to 1.084)
Central Asia	540 (508–575)	2.77 (2.61–2.94)	1239 (1070–1415)	3.67 (3.16–4.19)	−0.139 (−0.593 to 0.317)	51,031 (41,584–63,659)	286.47 (233.21–358.04)	177,463 (136,285–231,785)	522.52 (401.19–682.84)	1.403 (1.16 to 1.647)
Central Europe	1310 (1258–1366)	2.95 (2.84–3.08)	897 (822–978)	2.19 (2.02–2.38)	−1.302 (−1.516 to −1.087)	131,718 (107,249–161,947)	300.78 (244.94–369.78)	158,740 (118,407–209,890)	339.62 (254.58–447.66)	0.386 (0.282 to 0.49)
Central Latin America	4055 (3938–4179)	9.32 (9.03–9.61)	9677 (8699−10,767)	1.26 (1.13–1.4)	0.097 (−0.283 to 0.478)	353,399 (296,686–421,766)	838.46 (705.16–998.17)	985,677 (806,504–1,215,634)	1087.03 (889.2–1340.83)	0.679 (0.522 to 0.835)
Central Sub-Saharan Africa	1114 (847–1438)	9.21 (6.95–11.93)	2934 (2166–3874)	1.95 (1.79–2.12)	−0.249 (−0.35 to −0.149)	74,811 (59,398–93,280)	618.71 (491.09–773.6)	273,877 (213,573–351,684)	787.79 (614.56–1010.3)	0.73 (0.649 to 0.811)
East Asia	7909 (6957–9020)	2.02 (1.74–2.29)	8336 (6867−10,037)	0.8 (0.71–0.91)	−1.426 (−1.631 to −1.221)	1,175,181 (880,280–1,547,639)	297.07 (223.42–389.38)	2,739,719 (1,897,788–3,755,721)	491.13 (338.51–675.68)	1.619 (1.408 to 1.83)
Eastern Europe	1428 (1380–1479)	1.63 (1.58–1.69)	1768 (1625–1916)	0.81 (0.77–0.84)	−0.924 (−1.617 to −0.225)	156,469 (125,178–195,046)	204.26 (163.08–254.89)	281,215 (216,833–362,959)	343.97 (265.99–443.16)	0.868 (0.588 to 1.148)
Eastern Sub-Saharan Africa	3961 (3356–4659)	9.69 (8.32–11.49)	7834 (6600–9295)	2.07 (1.87–2.3)	−1.141 (−1.239 to −1.043)	238,739 (206,788–279,161)	585.78 (507.99–685.68)	600,723 (510,237–720,395)	554.29 (471.48–664.36)	−0.422 (−0.51 to −0.335)
High-income Asia Pacific	1547 (1404–1715)	2.29 (2.07–2.54)	575 (513–658)	3.9 (3.08–4.92)	−3.755 (−4.033 to −3.477)	199,622 (156,130–252,411)	298.32 (233.29–377.47)	317,469 (218,547–444,223)	467.28 (320.63–654.83)	1.379 (1.256 to 1.502)
High-income North America	3976 (3872–4089)	3.69 (3.59–3.8)	4532 (4360–4698)	8.67 (6.91–10.81)	−0.38 (−0.623 to −0.136)	341,855 (290,246–406,951)	327.28 (277.64–389.83)	692,254 (539,458–886,499)	550.48 (429.19–704.94)	1.58 (1.475 to 1.686)
North Africa and Middle East	2990 (2629–3455)	3.61 (3.16–4.2)	8133 (6815–9755)	3.6 (3.46–3.73)	0.07 (−0.028 to 0.168)	290,679 (236,025–360,007)	348.72 (283.57–431.54)	1,484,995 (1,100,705–1,976,778)	645.48 (478.49–859.42)	2.079 (1.986 to 2.172)
Oceania	431 (319–572)	25.33 (18.21–33.76)	1064 (832–1364)	10.64 (9.57–11.84)	−0.055 (−0.118 to 0.009)	26,838 (20,747–34,002)	1604.94 (1244.49–2032.82)	88,463 (70,751–110,147)	2061.26 (1650.17–2564.32)	0.754 (0.719 to 0.79)
South Asia	13,502 (12,118−14,858)	4.49 (4.04–4.95)	30,433 (26,654−34,256)	4.88 (4.66–5.11)	0.221 (0.186 to 0.256)	1,274,008 (1,051,955–1,538,098)	425.12 (351.81–511.68)	4,214,345 (3,270,834–5,386,334)	660.23 (513.32–842.63)	1.369 (1.301 to 1.438)
Southeast Asia	8524 (7506–9926)	6.63 (5.81–7.72)	16,793 (14,554−19,269)	3.56 (2.98–4.27)	−0.278 (−0.379 to −0.176)	599,258 (515,519–702,230)	469.81 (404.74–550.1)	1,477,327 (1,235,958–1,781,319)	561.85 (469.91–677.66)	0.176 (0.061 to 0.29)
Southern Latin America	489 (445–535)	3.01 (2.74–3.28)	519 (468–577)	4.83 (4.23–5.44)	−1.265 (−1.432 to −1.097)	39,733 (33,307–47,611)	253.23 (212.22–303.52)	83,716 (62,351–110,774)	335.09 (249.63–443.28)	0.889 (0.808 to 0.97)
Southern Sub-Saharan Africa	1001 (892–1126)	7.6 (6.81–8.65)	2254 (1973–2552)	8.7 (6.42–11.48)	0.802 (0.449 to 1.156)	70,888 (61,460–81,877)	535.35 (465.18–616.05)	182,398 (153,048–216,494)	664.11 (558.75–786.14)	0.962 (0.709 to 1.216)
Tropical Latin America	2917 (2802–3042)	6.58 (6.32–6.87)	4299 (4104–4503)	7.44 (6.26–8.83)	−1.277 (−1.466 to −1.087)	240,216 (207,591–279,328)	544.1 (469.84–633.02)	479,414 (386,493–591,563)	545.1 (439.56–672.61)	−0.112 (−0.233 to 0.01)
Western Europe	2202 (2126–2283)	1.59 (1.54–1.65)	1264 (1211–1315)	5.97 (4.69–7.28)	−2.265 (−2.414 to −2.116)	297,896 (230,766–379,609)	217.67 (168.57–277.48)	538,178 (369,827–743,499)	359.5 (246.17–498.02)	1.56 (1.481 to 1.639)
Western Sub-Saharan Africa	2505 (2099–2987)	5.54 (4.62–6.56)	6926 (5427–8448)	8.41 (7.36–9.52)	0.148 (0.03 to 0.266)	182,502 (152,795–217,228)	409.72 (343.95–486.71)	678,182 (547,008−850,828)	564.31 (456.71–706.58)	0.979 (0.908 to 1.049)

ASR = age-standardized rate, DALYs = disability-adjusted life years, EAPC = estimated annual percentage changes, SDI = sociodemographic index.

**Figure 1.**
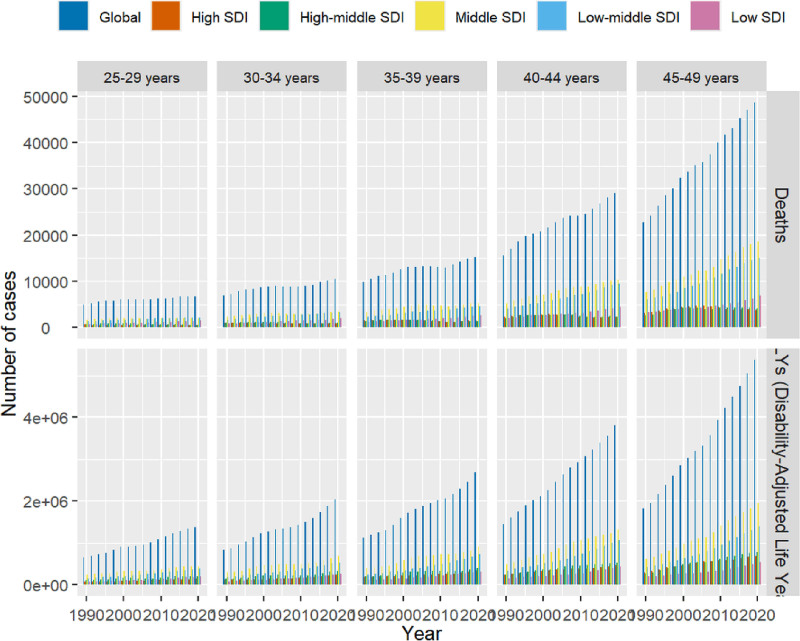
Disability-adjusted life years (DALYs), and death number of diabetes mellitus due to metabolic factors among young adults by age and sociodemographic index, from 1990 to 2021.

**Figure 2.**
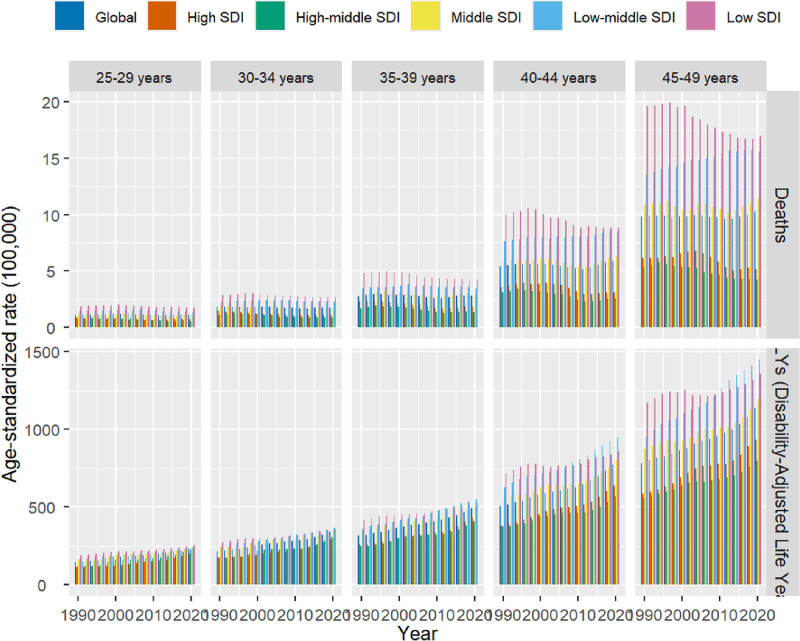
Age-specific disability-adjusted life years (DALYs), and death rates of diabetes mellitus due to metabolic factors among young adults by age and sociodemographic index, from 1990 to 2021.

In 2021, there were 15,754,105 DALYs of DM due to metabolic factors, with a 95% UI ranging from 12,318,727 to 20,033,528, representing a significant increase of 62.63% compared to the year 1990. The ASDR of DM due to metabolic factors in 2021 was recorded at a lower value of 569.45/100,000 (95% UI = 444.94–724.43) as opposed to the rate in the year 1990 which stood at 367.76/100,000 (95% UI = 304.68–444.97). Furthermore, the EAPC indicated an increasing trend in ASDR from 1990 to 2021 [1.308 (95% UI = 1.25–1.37)], leading to an overall rise in ASDR over this period. In 2021, the highest ASDR was observed in the 45 to 49 age group (1161.27 per 100,000, 95% UI = 940.03–1443.92). Notably, the fastest-growing age group was 25 to 29 years (EAPC = 1.48, 95% CI = 1.39–1.57). Males had a higher ASDR than females across all age groups (Figs. 1 and 2).

### 3.2. Regional level

In 2021, the 5 regions with the highest ASMR of DM due to metabolic were Andean Latin America, Oceania, Southern Sub-Saharan Africa, High-income North America, Western Sub-Saharan Africa (Table S1, Supplemental Digital Content, https://links.lww.com/MD/Q795). Among these regions, Andean Latin America exhibited the highest ASMR for DM due to metabolic at 25.18 per 100,000 population (95% UI = 19.69–32.27). Andean Latin America exhibited the highest ASMR for DM due to metabolic at 25.18 per 100,000 population (95% UI = 19.69–32.27). Oceania reported an ASMR of 10.64 per 100,000 population (95% UI = 9.57–11.84). The Southern Sub-Saharan Africa reported an ASMR of 8.7 per 100,000 population (95% UI = 6.42–11.48), the High-income North America exhibited the ASMR for DM due to metabolic at 8.67 per 100,000 population (95% UI = 6.91–10.81), and the Western Sub-Saharan Africa reported an ASMR of 8.41 per 100,000 population (95% UI = 7.36–9.52). Compared to 1990, the ASMR of DM due to metabolic factors showed a statistically significant decline in all regions in 2021.

Unlike ASMR, ASDR exhibits a different trend. The ASDR of DM due to metabolic factors showed a statistically significant increase in all 21 GBD regions compared to 1990. Oceania had the highest ASDR of DM due to metabolic factors, recorded at 2061.26 per 100,000 population (95% UI = 1650.17–2564.32). Central Latin America had the second-highest ASDR, recorded at 1087.03 per 100,000 population (95% UI = 889.2–1340.83), followed by High-income North America, with an ASDR of 1060.15 per 100,000 population (95% UI = 823.62–1366.63). Central Sub-Saharan Africa had an ASDR of 787.79 per 100,000 population (95% UI = 614.56–1010.3), while Sub-Saharan Africa had an ASDR of 664.11 per 100,000 population (95% UI = 558.75–786.14).

**Figure 3.**
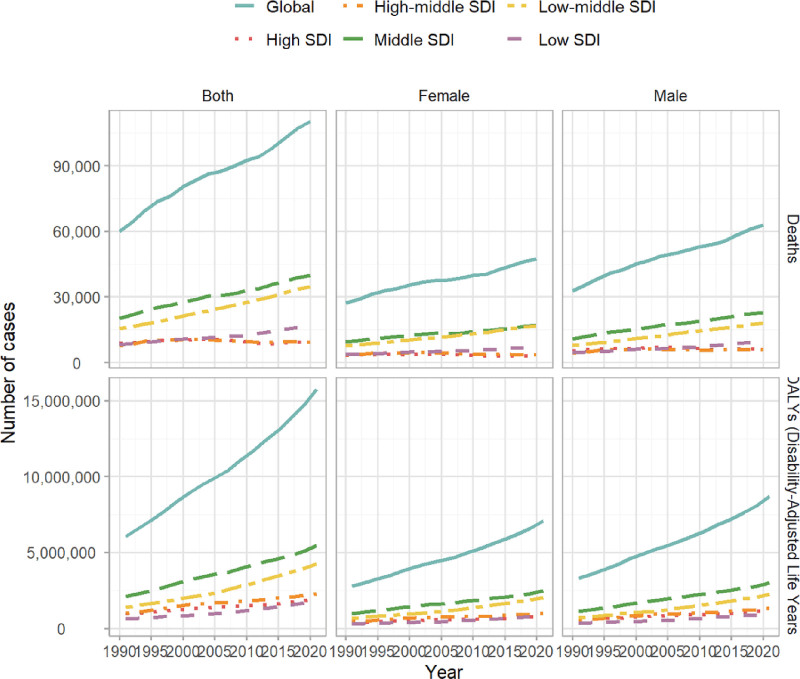
Temporal trends in disability-adjusted life years (DALYs), and death number of diabetes mellitus due to metabolic factors in young adults by sex and sociodemographic index, from 1990 to 2021.

**Figure 4.**
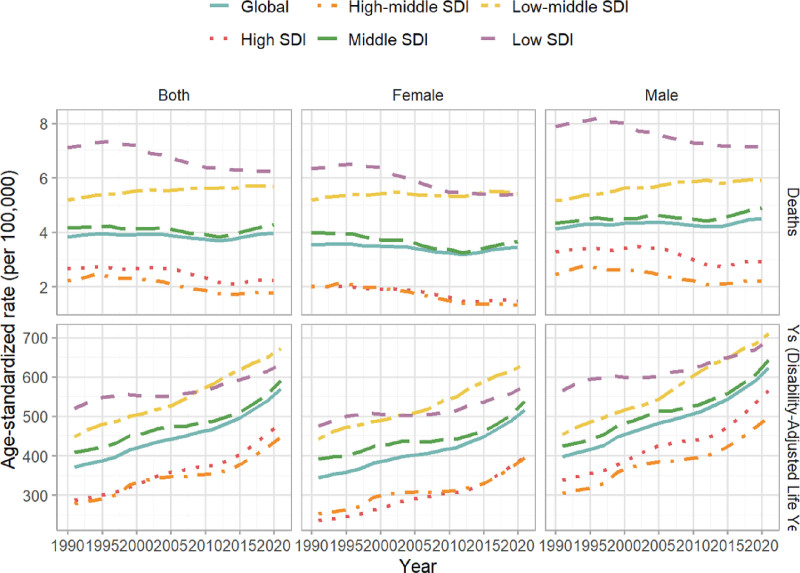
Temporal trends in age-specific disability-adjusted life years (DALYs), and death rates of diabetes mellitus due to metabolic factors in young adults by sex and sociodemographic index, from 1990 to 2021.

The global burden of DM due to metabolic factors in young adults shows significant regional variations closely linked to SDI levels. Regarding mortality, the age-standardized rate (ASR) per 100,000 population in 2021 was lowest in High-middle SDI regions, at 1.77 (95% UI = 1.61–1.94), followed by High-SDI regions at 2.19 (95% UI = 2.07–2.33). Middle SDI regions had a higher ASR of 4.34 (95% UI = 4.02–4.66), with a more pronounced burden in Low-middle SDI regions at 5.66 (95% UI = 5.06–6.31), peaking in Low-SDI regions, at 6.29 (95% UI = 5.45–7.33). Regarding DALYs, the lowest ASR was also observed in High-middle SDI regions, at 446.46 (95% UI = 320.34–602.98), followed by High-SDI regions at 483.12 (95% UI = 354.53–640.61). Middle SDI regions had a higher burden, with an ASR of 590.38 (95% UI = 461.91–749.41), while Low-middle SDI regions had the highest burden, at 671.97 (95% UI = 534.64–843.69). Low-SDI regions had a slightly lower, though still substantial, burden, at 633.32 (95% UI = 518.27–789.34) (Figs. 3 and 4).

### 3.3. National level

In 2021, the 3 countries with the highest ASMR of DM due to metabolic factors were Fiji [49.83 per 100,000 (95% UI = 36.01–67.53)], Kiribati [47.5 per 100,000 (95% UI = 31.89–70.06)], and the Marshall Islands [44.3 per 100,000 (95% UI = 26.46–69.66)] (Table S2, Supplemental Digital Content, https://links.lww.com/MD/Q795). India [22,180 (95% UI = 19,031–25,277)], Mexico [7058 (95% UI = 6229–7992)], and China [7613 (95% UI = 6121–9300)] had the highest death cases for DM due to metabolic (Additional file 1: Table S2, Supplemental Digital Content, https://links.lww.com/MD/Q795). From 1990 to 2021, Lesotho (EAPC = 4.58, 95% CI = 3.85 to 5.32), Zimbabwe (EAPC = 3.72, 95% CI = 2.69 to 4.76), and Mauritius (EAPC = 2.988, 95% CI = 2.38 to 3.60) experienced the strongest increase in the ASMR of DM due to metabolic. The Singapore (EAPC = −6.62, 95% CI = −7.24 to −6.00), Republic of Korea (EAPC = −4.845, 95% CI = −5.19 to −4.50), and Spain (EAPC = −4.07, 95% CI = −4.26 to −3.87) had the largest increase in the ASMR of DM due to metabolic.

In 2021, the top 3 countries with the highest ASDR of DM due to metabolic were Marshall Islands [2842.49/100,000 (95% UI = 2149.19–3725.36)], Fiji [3309.29/100,000 (95% UI = 2570.29–4301.04)], and Nauru [3024.6/100,000 (95% UI = 2244.96–3979.96)] (Table S2, Supplemental Digital Content, https://links.lww.com/MD/Q795). India [3,105,893 (95% UI = 2400,100–3984,133)], Mexico [654,443 (95% UI = 540,124–790,368)], and China [2637,045 (95% UI = 1818,792–3630,137)] had the highest DALYs for DM due to metabolic (Additional file 1: Table S2, Supplemental Digital Content, https://links.lww.com/MD/Q795). From 1990 to 2021, Lesotho (EAPC = 3.97, 95% CI = 3.46 to 4.48), Morocco (EAPC = 3.25, 95% CI = 3.16 to 3.33), and Libya (EAPC = 3.24, 95% CI = 3.06 to 3.42) had the largest increase in the ASDR of DM due to metabolic. Rwanda (EAPC = −2.62, 95% CI = −3.07 to −2.17), Maldives (EAPC = −1.34, 95% CI = −1.64 to −1.05), and Ethiopia (EAPC = −2.18, 95% CI = −2.40 to −1.97) experienced the strongest decrease in the ASDR of DM due to metabolic (Table S2, Supplemental Digital Content, https://links.lww.com/MD/Q795).

### 3.4. Projected DALYs and deaths

**Figure 5.**
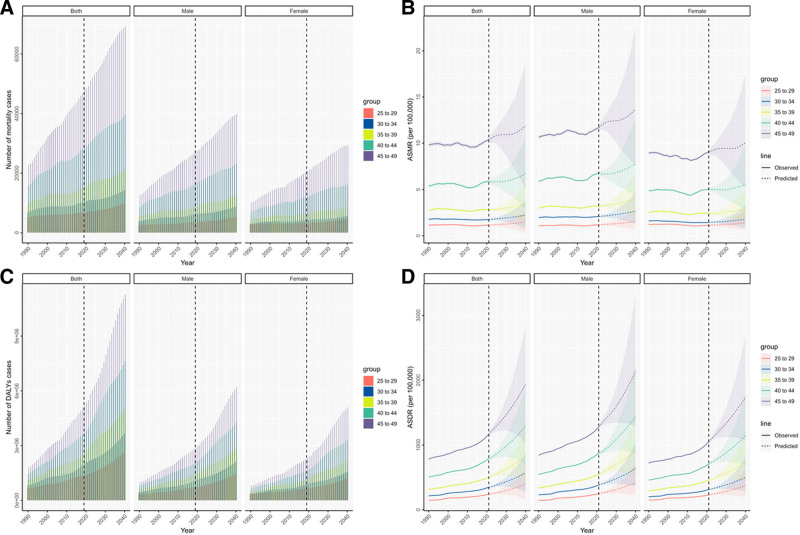
Temporal trends of the number and age-standardized rates for diabetes mellitus due to metabolic factors by sex from 2021 to 2040. (A) The number of death, (B) the age-standardized DALYs rates, (C) the number of DALYs, (D) the age-standardized mortality rates. DALYs = disability-adjusted life years.

**Figure 6.**
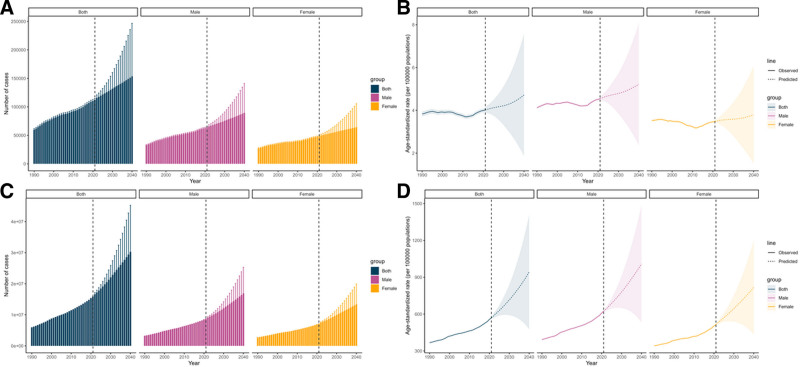
Temporal trends of the number and age-standardized rates for diabetes mellitus due to metabolic factors by age from 2021 to 2040. (A) The number of death, (B) the age-standardized DALYs rates, (C) the number of DALYs, (D) the age-standardized mortality rates. DALYs = disability-adjusted life years.

From 2021 to 2040, the ASMR of DM due to metabolic is projected to remain relatively stable, rising from 4.03 (95% CI = 3.93–4.12) per 100,000 in 2021 to 4.72 (95% CI = 1.85–7.58) per 100,000 in 2040. In contrast, the ASDR is projected to increase significantly, rising from 569.45 (95% CI = 568.22–570.68) per 100,000 in 2021 to 942.97 (95% CI = 475.42–1410.53) per 100,000 in 2040 (Figs. 5 and 6). Notably, the percentage increase in males exceeds that in females. Across both sexes, ASDR is projected to rise, increasing from 622.64 to 1008.67 per 100,000 in males and from 515.44 to 817.76 per 100,000 in females by 2040. Over the forecast period, all age groups are projected to show a continuous increase in ASDR, with the 45 to 49 age group experiencing the largest increase, rising from 1161.29 to 2132.71 per 100,000 population (Table S3, Supplemental Digital Content, https://links.lww.com/MD/Q795).

### 3.5. Frontier analysis

**Figure 7.**
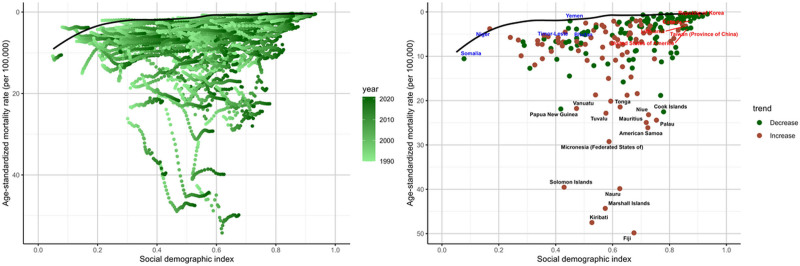
Frontier analysis based on the age-standardized rates of mortality for diabetes mellitus due to metabolic factors from 1990 to 2021 and specifically in 2021 by SDI. SDI = sociodemographic index.

**Figure 8.**
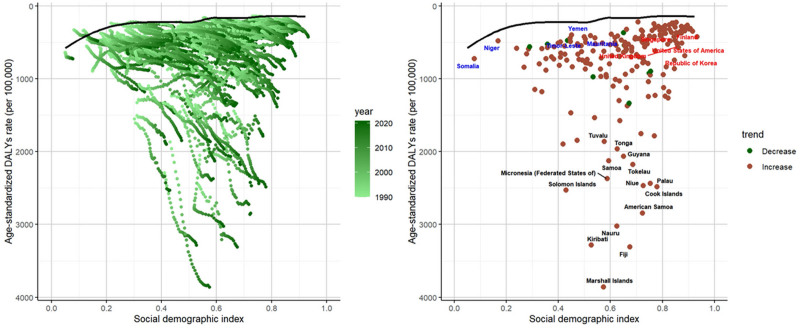
Frontier analysis based on the age-standardized rates of DALYs for diabetes mellitus due to metabolic factors from 1990 to 2021 and specifically in 2021 by SDI. DALYs = disability-adjusted life years, SDI = sociodemographic index.

The effective difference from the frontier for each country or territory was calculated based on the 2021 DALYs and SDI. Overall, the effective difference for a given SDI tended to be smaller and showed lower variance as SDI increased. The top 10 countries with the smallest deviation in DALYs from the frontier were Albania, Australia, Austria, Denmark, France, Greenland, Romania, Slovakia, Slovenia, and Somalia (range: 59.69–130.49). In contrast, the top 10 countries with the largest deviation in DALYs from the frontier were American Samoa, Cook Islands, Fiji, Kiribati, Marshall Islands, Micronesia, Nauru, Niue, Palau, and Solomon Islands (range: 2207.89–3694.94). The top 10 countries with the smallest deviation in mortality from the frontier were Albania, Iceland, Ireland, Japan, Luxembourg, Niger, San Marino, Singapore, Somalia, Spain, Switzerland, and Yemen (range: 0–0.14) (Figs. 7 and 8). In contrast, the top 10 countries with the largest deviation in mortality from the frontier were American Samoa, Fiji, Kiribati, Marshall Islands, Mauritius, Micronesia, Nauru, Niue, Palau, and Solomon Islands (range: 22.50–49.10) (Tables S4 and S5, Supplemental Digital Content, https://links.lww.com/MD/Q795).

## 4. Discussion

Our study provides the first comprehensive analysis of the global burden of DM due to metabolic factors in young adults from 1990 to 2021, highlighting trends across regions and sociodemographic indices. We observed significant regional variations closely linked to SDI levels, with a decline in ASMR but a rising ASDR, indicating an increasing disease burden despite reduced mortality. In young adults aged 25 to 49 years, ASDR showed a consistent upward trend globally and across all SDI categories. The BAPC model predicts a continued rise in mortality and DALYs over the next 2 decades, while frontier analysis reveals substantial potential for improvement across all levels of development. These findings underscore the urgent need for targeted interventions to mitigate the growing burden of DM due to metabolic factors.

This study highlights the interplay of demographic and metabolic factors. Total mortality rose by 86.81%, and DALYs increased by 62.63% from 1990 to 2021, largely reflecting population growth and increased exposure to metabolic risk factors such as obesity, hyperglycemia, and dyslipidemia.^[7]^ However, a contrasting trend emerges with a decrease in ASMR but an increase in ASDR. This paradox underscores the complexity of diabetes-related outcomes. The decline in ASMR suggests improvements in diabetes care, including early detection, enhanced pharmacological management, and reduced case fatality rates for complications such as cardiovascular and renal diseases.^[12]^ Despite these advancements, the rising ASDR indicates an increasing prevalence of diabetes-related disability. This could stem from prolonged survival with diabetes, leading to a higher burden of complications such as neuropathy, retinopathy, and amputations, which contribute significantly to DALYs.^[13]^ Furthermore, the shift towards earlier disease onset exacerbates the cumulative lifetime risk of complications, amplifying the overall disability burden.

This study found significant age disparities in the burden of DM due to metabolic factors. Individuals aged 45 to 49 years exhibited the highest ASMR and ASDR, likely due to cumulative metabolic risks, while the 25 to 29 age group showed the fastest-growing burden, reflecting the rising prevalence of early-onset obesity and metabolic syndrome.^[14]^ These trends highlight a need for intensified prevention efforts targeting younger population to curb the trajectory of diabetes-related disabilities. The consistently higher ASMR and ASDR in males across all age groups emphasize sex-specific differences, potentially driven by greater visceral fat accumulation, poorer glycemic control, and health-seeking behaviors compared to females.^[15]^ These findings align with prior GBD studies.

The observed negative correlation between ASMR/ASDR and SDI reflects the disparity in healthcare access, public health infrastructure, and resource allocation. Higher SDI regions exhibit lower ASMR and ASDR, which can be attributed to effective management of metabolic factors, advanced healthcare systems, better disease awareness, and widespread implementation of prevention and management strategies.^[16]^ For example, early screening and control of specific metabolic factors such as blood glucose levels, lipid profiles, and body weight, along with the integration of innovative pharmacological treatments like GLP-1 receptor agonists and SGLT-2 inhibitors, have substantially reduced mortality rates.^[17–19]^ On the other hand, the burden in lower SDI regions, although higher, is starting to show notable progress. Particularly, the rapid decline in ASMR in low-SDI regions, second only to high-SDI regions, highlights the success of targeted health policies and global initiatives.^[20]^ Despite progress in reducing mortality, ASDR demonstrates an alarming global upward trend, particularly in high-SDI regions. High-SDI regions exhibited the fastest ASDR growth due to inadequate management of key metabolic factors, including obesity, hyperglycemia, dyslipidemia, and sedentary lifestyles.^[21]^ Furthermore, advances in DM management, while reducing mortality, prolong survival, leading to an accumulation of patients with long-term complications such as neuropathy, nephropathy, and cardiovascular diseases.^[22]^

In 2021, countries with the highest ASMRs (e.g., Fiji, Kiribati, and Marshall Islands) and ASDRs (e.g., Marshall Islands, Fiji, and Nauru) are predominantly small island nations, characterized by limited healthcare infrastructure, high prevalence of obesity, and poor access to DM care. Traditional dietary patterns have shifted to high-calorie, processed foods due to globalization, exacerbating metabolic risks.^[23]^ These findings align with prior research linking rapid urbanization, sedentary lifestyles, and genetic predispositions in Pacific Island population to their disproportionate DM burden.^[24]^ Conversely, populous countries like India and China contribute the largest absolute number of DM deaths and disability, reflecting their high population sizes and growing DM prevalence due to aging population and lifestyle transitions. Countries such as Singapore, South Korea, and Spain experienced the most significant ASMR declines, largely attributable to advancements in healthcare systems, robust preventive policies, and widespread adoption of innovative therapies, such as SGLT-2 inhibitors and GLP-1 receptor agonists. These nations have also emphasized early screening, glycemic control, and cardiovascular risk management. Singapore’s integrated health policies and South Korea’s universal healthcare are particularly effective models.^[25]^ In contrast, countries like Lesotho and Zimbabwe experienced sharp increases in ASMR, likely driven by limited healthcare access, insufficient preventive measures, and the growing influence of urbanization and westernized diets.^[26]^ The weak health systems in sub-Saharan Africa often fail to address DM complications effectively, contributing to high mortality rates.

Frontier analysis revealed that as SDI increased, the differences in DALYs and mortality rates diminished, showing less variability. However, particular attention should be paid to low-SDI frontier countries, which demonstrated remarkable outcomes despite their limited resources. This analysis highlights that low-SDI countries such as Somalia, Niger and Albania have managed the burden of these diseases exceptionally well. Despite facing resource constraints, these nations serve as notable examples by effectively implementing strategies such as community-based health interventions and strengthening primary healthcare systems, offering valuable insights for other nations facing similar challenges. In contrast, several high-SDI countries, such as the Mauritius and Trinidad and Tobago, have shown suboptimal performance, suggesting that these nations often struggle to implement universal and equitable health strategies despite having more resources. Therefore, Future research should prioritize identifying the key factors driving success in these exemplary countries and addressing the barriers that hinder progress in nations with poorer outcomes.

These data have some limitations. Our study relied extensively on GBD estimates, which inherently share their constraints. The accuracy of these estimates depends on the quality and comprehensiveness of each nation’s vital registration systems, which vary across regions. To address these shortcomings, modeling techniques were employed to evaluate the global disease burden. Additionally, our analysis of DM due to metabolic factors utilized country-level rather than patient-level data, which could result in underestimating or overestimating the associations. Moreover, the classification criteria and disease definitions in the GBD database may not align with those used in other international datasets or research, potentially affecting the reliability of burden estimations. Finally, variations in medical practices, diagnostic advancements, and reporting standards across different regions and time periods could introduce biases into the interpretation of long-term trends.

## 5. Conclusion

In summary, metabolic factors play a significant role in contributing to the burden of DM. From 1990 to 2021, both the age-standardized and absolute burden of DM due to metabolic factors increased substantially on a global scale, and this trend is projected to continue rising through 2040. Effective management of metabolic factors, particularly in high-risk population, will be crucial in mitigating the projected increase in both the prevalence and complications of diabetes, guiding public health decisions for the future.

## Author contributions

**Conceptualization:** Guokun Sun, Yating Wen, Xiaojing Han, Qiao Wang, Yi Mao, Xiaoqing Han.

**Data curation:** Guokun Sun, Yating Wen, Xiaojing Han, Qiao Wang, Yunli Wang.

**Formal analysis:** Guokun Sun, Qiao Wang, Xiaolu Sun.

**Investigation:** Guokun Sun, Yating Wen, Xiaojing Han, Yi Mao, Xiaolu Sun.

**Methodology:** Yating Wen, Xiaojing Han, Qiao Wang, Yunli Wang, Xiaolu Sun, Yan Zhai, Yaru Wen, Xiaoqing Han.

**Project administration:** Qiao Wang, Yunli Wang, Yaru Wen, Xiaoqing Han.

**Resources:** Guokun Sun, Xiaojing Han, Qiao Wang, Yi Mao, Yan Zhai, Yaru Wen, Xiaoqing Han.

**Software:** Guokun Sun, Yating Wen, Xiaojing Han, Qiao Wang, Yunli Wang, Yi Mao, Xiaolu Sun, Yan Zhai, Yaru Wen, Xiaoqing Han.

**Supervision:** Qiao Wang, Yunli Wang, Yi Mao, Xiaolu Sun, Yan Zhai.

**Validation:** Yating Wen, Yunli Wang, Yan Zhai, Xiaoqing Han.

**Visualization:** Xiaoqing Han.

**Writing – original draft:** Guokun Sun, Yating Wen, Xiaojing Han.

**Writing – review & editing:** Xiaoqing Han.

## Supplementary Material
