# Supplementary material for: Global, regional, and national burden of diabetes mellitus due to metabolic factors in young adults, 1990 to 2021 and predictions to 2040: An analysis of the Global Burden of Disease Study 2021
Source: Medicine (Baltimore). 2025 Dec 12;104(50):e46261. doi: 10.1097/MD.0000000000046261 (PMC12708187; doi:10.1097/MD.0000000000046261)
Supplement: Supplementary file 1 [file medi-104-e46261-s001.docx]

**Supplementary Table 1.** Mortality and DALY rates of diabetes mellitus due to metabolic factors in young adults (aged 25-49 years) by age and sex group in 1990 and 2021, and average annual percentage changes from 1990 to 2021

|  | **Mortality** | | | | | **DALYs** | | | | |
| --- | --- | --- | --- | --- | --- | --- | --- | --- | --- | --- |
| **Location** | **Num_1990 (95% UI)** | **ASR_1990 per 100,000 persons (95% UI)** | **Num_2021 (95% UI)** | **ASR_2021 per 100,000 persons (95% UI)** | **EAPC (95% CI)** | **Num_1990 (95% UI)** | **ASR_1990 per 100,000 persons (95% UI)** | **Num_2021 (95% UI)** | **ASR_2021 per 100,000 persons (95% UI)** | **EAPC (95% CI)** |
| **Sex** |  |  |  |  |  |  |  |  |  |  |
| Male | 32725(30493-35148) | 4.12(3.84-4.43) | 63779(57902-69762) | 4.55(4.12-4.98) | 0.131  (0.058 to 0.204) | 3179214(2615373-3848709) | 362.61(323.72-474.28) | 8682365(6787813-11033547) | 622.64(486.76-791.59) | 1.404  (1.352 - 1.455) |
| Female | 27197(25313-30133) | 3.53(2.89-3.90) | 52562(44124-52562) | 3.48(3.20-3.81) | -0.246  (-0.356 to -0.137) | 2688609(2227122-3268815) | 341.96(283.79-414.87) | 9021787(5474017-9021787) | 515.44(398.74-657.81) | 1.2  (1.126- 1.275) |
| **Age (years)** |  |  |  |  |  |  |  |  |  |  |
| 25-29 | 4994(4709-5357) | 1.13(1.07-1.21) | 6816(6301-7403) | 1.16(1.07-1.26) | -0.154  (-0.282 to -0.025) | 657050(527175-818904) | 148.45(119.10-185.01) | 1399558(1042362-1823333) | 237.88(177.17-309.91) | 1.482  (1.39 - 1.574) |
| 30-34 | 6886(6525-7325) | 1.79(1.69-1.90) | 10647(9823-11513) | 1.76(1.63-1.90) | -0.209  (-0.261 to -0.158) | 834254(679975-1034294) | 216.45(176.42-268.35) | 2099566(1574746-2772315) | 347.33(260.51-458.63) | 1.438  (1.364 - 1.513) |
| 35-39 | 9808(9327-10421) | 2.78(2.65-2.96) | 15610(14493-16743) | 2.78(2.58-2.99) | -0.207  (-0.319 to -0.094) | 1114907(903870-1381771) | 316.52(256.60-392.28) | 2809938(2136656-3660526) | 500.99(380.96-652.66) | 1.397  (1.343 - 1.451) |
| 40-44 | 15465(14602-16380) | 5.40(5.10-5.72) | 29639(27447-31932) | 5.92(5.49-6.38) | 0.02  (-0.12 to 0.161) | 1444323(1198579-1727842) | 504.16(418.38-603.13) | 3946391(3113878-4940343) | 788.88(622.46-987.57) | 1.315  (1.236- 1.395) |
| 45-49 | 22768(21651-24289) | 9.80(9.32-10.46) | 49229(45624-53169) | 10.40(9.64-11.23) | 0.039  (-0.03 to 0.109) | 1816291(1540386-2151428) | 782.23(663.40-926.56) | 5498650(4451083-6837008) | 1161.27(940.03-1443.92) | 1.16  (1.096 - 1.224) |

**EAPC: estimated annual percentage changes ;**

**ASR: age-standardized rate;**

**DALYs: disability-adjusted life years.**

**Supplementary Table 2.** Mortality and DALY rates of diabetes mellitus due to metabolic factors in young adults (aged 25-49 years) by 204 regions/countries in 1990 and 2021, and average annual percentage changes from 1990 to 2021

|  | **Mortality** | | | | | **DALYs** | | | | |
| --- | --- | --- | --- | --- | --- | --- | --- | --- | --- | --- |
| **Location** | **Num_1990 (95% UI)** | **ASR_1990 per 100,000 persons (95% UI)** | **Num_2021 (95% UI)** | **ASR_2021 per 100,000 persons (95% UI)** | **EAPC (95% CI)** | **Num_1990 (95% UI)** | **ASR_1990 per 100,000 persons (95% UI)** | **Num_2021 (95% UI)** | **ASR_2021 per 100,000 persons (95% UI)** | **EAPC (95% CI)** |
| Afghanistan | 130(84-204) | 5.7(3.69-8.9) | 565(351-897) | 7.22(4.49-11.45) | 0.763  (0.695 to 0.831) | 12395(9027-16796) | 565.14(412.87-763.87) | 91942(65063-125373) | 1179.81(836.9-1606.04) | 2.478  (2.439 to 2.518) |
| Albania | 8(6-11) | 0.8(0.6-1.07) | 6(4-9) | 0.68(0.48-0.96) | -0.438  (-0.666 to -0.21) | 1521(1088-2100) | 159.83(113.2-222.12) | 1971(1336-2812) | 221.59(150.21-315.69) | 1.066  (0.991 to 1.141) |
| Algeria | 101(71-145) | 1.87(1.31-2.67) | 370(262-509) | 2.28(1.62-3.14) | 0.926  (0.798 to 1.053) | 15806(11646-21216) | 281.13(207.31-376.9) | 100331(70260-137206) | 614.83(430.39-841.09) | 2.554  (2.524 to 2.584) |
| American Samoa | 2(2-3) | 18.82(13.63-25.5) | 5(3-6) | 26.12(18.86-35.23) | 1.071  (0.715 to 1.428) | 193(149-244) | 1499.39(1160.44-1891.34) | 487(370-638) | 2842.49(2149.19-3725.36) | 2.025  (1.763 to 2.289) |
| Andorra | 0(0-1) | 1.76(1.14-2.6) | 0(0-1) | 1.03(0.61-1.52) | -1.468  (-1.69 to -1.245) | 52(38-69) | 226.95(165.89-303.64) | 129(89-179) | 349.96(240.93-488.14) | 1.424  (1.339 to 1.508) |
| Angola | 209(142-299) | 8.92(6.08-12.82) | 622(416-886) | 8.22(5.49-11.72) | -0.278  (-0.403 to -0.153) | 15465(11424-20121) | 640.51(472.25-835.45) | 65245(48860-86085) | 835.76(626.82-1102.28) | 0.927  (0.83 to 1.025) |
| Antigua and Barbuda | 2(2-2) | 11.47(10.13-12.94) | 2(2-3) | 6.01(5.21-6.93) | -1.487  (-1.739 to -1.235) | 166(140-200) | 924.62(782.79-1117.39) | 330(245-437) | 922.8(684.32-1220.15) | 0.191  (0.069 to 0.312) |
| Argentina | 354(315-396) | 3.38(3-3.78) | 407(358-465) | 2.42(2.12-2.76) | -1.173  (-1.36 to -0.985) | 28423(23893-33834) | 271.11(227.9-322.74) | 58633(44284-77412) | 348.95(263.56-460.6) | 0.762  (0.659 to 0.866) |
| Armenia | 36(33-38) | 3.73(3.45-4.01) | 37(31-44) | 3.34(2.8-3.97) | -0.602  (-1.136 to -0.065) | 3431(2827-4214) | 349.96(287.63-431.2) | 5002(3828-6614) | 450.09(344.99-594.68) | 0.516  (0.231 to 0.802) |
| Australia | 85(76-95) | 1.36(1.22-1.52) | 117(103-132) | 1.25(1.1-1.41) | -0.731  (-0.972 to -0.489) | 10337(8198-13076) | 165.27(131.13-209.05) | 21684(15834-29257) | 232.73(170.01-313.87) | 1.045  (0.962 to 1.127) |
| Austria | 48(43-53) | 1.65(1.47-1.84) | 21(19-24) | 0.64(0.57-0.73) | -2.469  (-2.791 to -2.145) | 4653(3763-5767) | 162.6(131.44-201.67) | 7203(4941-10093) | 224.26(153.66-313.99) | 1.119  (1.035 to 1.202) |
| Azerbaijan | 61(48-76) | 3.33(2.62-4.14) | 127(88-167) | 3.19(2.2-4.19) | -0.886  (-1.232 to -0.54) | 5608(4506-6991) | 296.44(237.87-369.5) | 18321(13631-24499) | 454.58(338.34-608.67) | 0.882  (0.687 to 1.078) |
| Bahamas | 9(7-10) | 11.23(9.77-12.75) | 14(11-18) | 9.24(7.06-12.08) | -0.974  (-1.166 to -0.782) | 698(580-848) | 890.64(741.45-1083.29) | 1690(1291-2196) | 1121.39(857.01-1456.12) | 0.529  (0.424 to 0.635) |
| Bahrain | 11(9-14) | 7.34(5.67-9.21) | 55(41-70) | 7.02(5.3-8.98) | -0.604  (-0.883 to -0.324) | 983(776-1231) | 605.09(479.37-756.84) | 7325(5468-9687) | 950.01(709.05-1256.22) | 1.176  (1.025 to 1.327) |
| Bangladesh | 1312(964-1715) | 5.33(3.94-6.94) | 2726(1915-3824) | 4.89(3.43-6.86) | 0.043  (-0.127 to 0.212) | 120316(93074-151599) | 471.72(366.22-591.92) | 420258(315114-560564) | 743.69(558.12-991.28) | 1.683  (1.586 to 1.779) |
| Barbados | 10(9-11) | 12.27(10.93-13.76) | 10(7-13) | 8.67(6.52-11.45) | -1.232  (-1.47 to -0.995) | 755(642-893) | 929.48(790.34-1099.66) | 1143(871-1495) | 1037.71(791.56-1355.18) | 0.14  (0.002 to 0.278) |
| Belarus | 51(46-57) | 1.48(1.32-1.65) | 74(58-92) | 2.1(1.66-2.6) | -1.031  (-1.923 to -0.132) | 6076(4780-7783) | 176.33(138.35-226.7) | 10018(7682-13228) | 279.73(215.22-368.06) | 0.478  (0.113 to 0.844) |
| Belgium | 46(42-52) | 1.32(1.18-1.47) | 26(23-29) | 0.63(0.56-0.71) | -2.861  (-3.156 to -2.565) | 7915(5860-10485) | 224.16(165.92-297.09) | 14381(9568-20786) | 367.86(244.68-530.18) | 1.56  (1.447 to 1.673) |
| Belize | 3(3-4) | 8.96(7.95-10.01) | 15(13-18) | 10.89(9.21-12.8) | 0.462  (0.008 to 0.918) | 284(238-340) | 705.99(593.06-847.88) | 1576(1264-1992) | 1100.51(883.08-1392.28) | 1.314  (1.028 to 1.6) |
| Benin | 45(33-61) | 4.73(3.42-6.35) | 172(116-246) | 5.57(3.76-7.97) | 0.374  (0.175 to 0.574) | 4058(3110-5157) | 400.95(308.41-508.92) | 22707(16742-30522) | 699.03(516.89-940.68) | 1.628  (1.547 to 1.709) |
| Bermuda | 1(1-1) | 4.88(4.27-5.58) | 1(0-1) | 2.39(1.88-2.99) | -2.502  (-2.733 to -2.271) | 108(88-134) | 448.88(366.47-554.64) | 125(90-169) | 523.39(376.91-708.64) | 0.389  (0.233 to 0.545) |
| Bhutan | 6(4-9) | 4.58(3.01-6.47) | 10(7-16) | 3.87(2.42-5.9) | -0.797  (-0.883 to -0.71) | 585(437-761) | 408.68(306.07-530.42) | 1502(1098-2043) | 538.77(394.89-733.58) | 0.751  (0.687 to 0.814) |
| Bolivia (Plurinational State of) | 100(73-135) | 6.37(4.67-8.6) | 205(140-293) | 5.27(3.59-7.53) | -1.001  (-1.197 to -0.804) | 7148(5536-9157) | 446.11(345.41-572.11) | 20840(15695-27876) | 529.47(398.67-708.47) | 0.324  (0.194 to 0.454) |
| Bosnia and Herzegovina | 48(39-58) | 3.11(2.56-3.79) | 32(22-43) | 2.57(1.76-3.44) | -0.6  (-0.782 to -0.417) | 4540(3633-5716) | 296.45(236.54-374.29) | 5147(3697-7153) | 406.55(293.55-562.37) | 0.998  (0.905 to 1.091) |
| Botswana | 15(10-24) | 5.73(3.65-8.79) | 41(27-59) | 4.81(3.2-6.91) | -0.575  (-0.915 to -0.235) | 1116(800-1532) | 398.76(284.77-549.19) | 4129(3081-5465) | 469.64(350.6-622.01) | 0.48  (0.288 to 0.672) |
| Brazil | 2822(2707-2944) | 6.63(6.36-6.92) | 4126(3926-4324) | 4.81(4.58-5.04) | -1.368  (-1.567 to -1.168) | 235933(203802-274058) | 547.21(472.29-635.95) | 462813(372164-572179) | 541.05(435.18-668.86) | -0.165  (-0.293 to -0.037) |
| Brunei Darussalam | 12(9-15) | 14.62(11.11-18.97) | 20(15-26) | 10.35(7.81-13.33) | -0.865  (-1.115 to -0.614) | 784(621-971) | 950.14(751.67-1178.16) | 2421(1822-3146) | 1237.46(931.27-1608.09) | 0.99  (0.732 to 1.248) |
| Bulgaria | 113(101-127) | 3.65(3.25-4.07) | 93(77-111) | 3.49(2.89-4.16) | -0.529  (-0.789 to -0.267) | 10535(8570-13002) | 337.09(274.96-415.09) | 11781(9044-15433) | 437.06(337.8-568.77) | 0.698  (0.582 to 0.814) |
| Burkina Faso | 141(101-195) | 7.19(5.14-9.99) | 347(230-491) | 6.74(4.45-9.55) | -0.194  (-0.332 to -0.057) | 9193(6965-12013) | 460.99(348.89-602.94) | 31788(23880-41692) | 593.55(446.04-778.33) | 0.801  (0.667 to 0.935) |
| Burundi | 122(80-178) | 10.65(6.96-15.52) | 236(149-371) | 7.55(4.78-11.83) | -1.67  (-1.893 to -1.446) | 7588(5476-10378) | 632.95(455.56-867.67) | 18209(13176-25426) | 559.91(404.04-783.61) | -0.848  (-1.03 to -0.666) |
| Cabo Verde | 1(1-2) | 2.36(1.72-3.09) | 8(5-11) | 4.15(2.81-5.99) | 1.632  (1.069 to 2.198) | 190(143-253) | 294.07(222.87-390.25) | 1232(899-1668) | 615.87(450.62-831.71) | 2.394  (2.074 to 2.715) |
| Cambodia | 157(113-219) | 6.89(4.94-9.64) | 320(209-485) | 5.95(3.87-9.01) | -0.563  (-0.672 to -0.453) | 10228(7782-13435) | 436.76(332.22-574.81) | 28601(20714-37929) | 518.7(374.74-687.91) | 0.455  (0.344 to 0.566) |
| Cameroon | 155(109-215) | 6.91(4.85-9.56) | 682(431-1016) | 8.74(5.53-13.03) | 0.552  (0.248 to 0.856) | 10981(8319-14346) | 474.35(359.05-620.39) | 60013(43907-80362) | 735.61(537.04-986.34) | 1.217  (0.995 to 1.439) |
| Canada | 225(203-249) | 2.14(1.93-2.36) | 272(247-300) | 2.1(1.9-2.31) | -0.72  (-1.037 to -0.401) | 19921(16599-23950) | 188.36(156.94-226.59) | 48304(35307-64597) | 370.93(271.73-495.35) | 1.681  (1.504 to 1.859) |
| Central African Republic | 80(56-111) | 13.06(9.03-18.14) | 181(112-269) | 12.71(7.85-18.89) | -0.228  (-0.348 to -0.109) | 5509(4150-7184) | 869.46(653.7-1135.43) | 16723(12136-22237) | 1148.92(833.77-1528.55) | 0.837  (0.76 to 0.915) |
| Chad | 49(34-69) | 3.98(2.77-5.63) | 198(133-293) | 5.75(3.86-8.54) | 1.102  (0.75 to 1.456) | 4234(3254-5522) | 334.15(256.69-436) | 20805(15589-27466) | 578.88(433.68-764.73) | 1.71  (1.477 to 1.944) |
| Chile | 96(86-106) | 2.29(2.06-2.55) | 87(77-98) | 1.25(1.11-1.4) | -1.747  (-1.977 to -1.516) | 9352(7450-11588) | 222.58(176.96-275.94) | 21062(14972-28860) | 302.46(215.03-414.27) | 1.076  (0.98 to 1.172) |
| China | 7177(6150-8202) | 1.95(1.67-2.23) | 7613(6121-9300) | 1.35(1.09-1.65) | -1.467  (-1.665 to -1.268) | 1123420(838306-1484366) | 294.09(220.35-386.73) | 2637045(1818792-3630137) | 490.53(336.57-677.56) | 1.667  (1.45 to 1.884) |
| Colombia | 351(314-389) | 3.89(3.48-4.31) | 427(345-519) | 2.45(1.98-2.98) | -2.362  (-2.734 to -1.989) | 43151(33892-56111) | 467.92(367.39-609.86) | 94170(67737-128704) | 538.69(387.56-736.42) | -0.043  (-0.263 to 0.178) |
| Comoros | 8(5-12) | 8.51(4.98-12.32) | 18(11-26) | 7.51(4.68-10.72) | -0.805  (-1.155 to -0.455) | 568(389-762) | 560.32(385.92-752.55) | 1609(1194-2138) | 664.63(492.91-883.31) | 0.317  (0.094 to 0.541) |
| Congo | 73(49-100) | 14.39(9.82-19.92) | 204(134-307) | 11.74(7.7-17.7) | -1.008  (-1.235 to -0.78) | 4522(3280-5972) | 869.22(631.01-1148.54) | 16489(12064-22110) | 943.26(689.67-1265.18) | -0.024  (-0.202 to 0.155) |
| Cook Islands | 1(1-2) | 27.51(19.77-36.75) | 1(1-2) | 22.53(15.94-31.59) | -0.546  (-0.675 to -0.417) | 107(82-136) | 1966.62(1506.5-2501.16) | 144(109-189) | 2482.81(1863.9-3261.78) | 0.752  (0.718 to 0.786) |
| Costa Rica | 20(18-23) | 2.61(2.31-2.91) | 50(43-58) | 2.91(2.47-3.36) | -0.461  (-1.097 to 0.18) | 3248(2428-4316) | 390.26(293.25-517.05) | 11643(8383-15950) | 666.79(480.33-913.67) | 1.586  (1.471 to 1.702) |
| Coted'Ivoire | 146(103-203) | 5.53(3.92-7.75) | 505(331-744) | 6.77(4.45-10) | 0.46  (0.179 to 0.743) | 11887(9028-15420) | 429.42(325.87-558.91) | 51666(38315-68340) | 665.2(493.34-879.48) | 1.285  (1.105 to 1.465) |
| Croatia | 42(37-47) | 2.35(2.1-2.63) | 21(18-25) | 1.4(1.17-1.61) | -1.523  (-1.711 to -1.335) | 4639(3636-5913) | 259.86(203.77-330.92) | 4654(3308-6492) | 298.38(213.06-414.73) | 0.501  (0.442 to 0.561) |
| Cuba | 145(130-161) | 3.93(3.53-4.36) | 67(56-80) | 1.57(1.31-1.87) | -3.962  (-4.568 to -3.353) | 16695(13280-21171) | 451.41(359.04-572.4) | 21450(14924-29916) | 523.72(363.25-730.28) | 0.059  (-0.175 to 0.294) |
| Cyprus | 10(7-13) | 3.52(2.71-4.59) | 10(7-13) | 1.7(1.25-2.25) | -2.872  (-3.221 to -2.522) | 855(664-1081) | 309.57(240.33-391.37) | 2066(1480-2813) | 360.25(257.83-490.72) | 0.116  (-0.011 to 0.243) |
| Czechia | 95(86-106) | 2.41(2.17-2.67) | 72(59-85) | 1.65(1.37-1.95) | -0.565  (-0.943 to -0.186) | 10609(8253-13604) | 266.89(208.3-341.37) | 14637(10492-20388) | 323.57(234.25-446.92) | 0.914  (0.77 to 1.058) |
| Democratic People's Republic of Korea | 218(143-328) | 3.14(2.06-4.73) | 359(231-559) | 3.37(2.17-5.27) | 0.162  (0.115 to 0.209) | 22372(16382-29434) | 323.13(236.63-425.11) | 52852(38102-71854) | 507.98(365.69-690.95) | 1.4  (1.373 to 1.426) |
| Democratic Republic of the Congo | 678(465-946) | 8.55(5.87-11.94) | 1821(1209-2604) | 8.23(5.45-11.77) | -0.164  (-0.303 to -0.025) | 46899(34920-60977) | 570.64(424.06-744.59) | 166170(124984-219538) | 727.57(547.57-961.24) | 0.717  (0.603 to 0.831) |
| Denmark | 49(44-55) | 2.42(2.14-2.72) | 19(17-22) | 0.9(0.79-1.03) | -2.982  (-3.477 to -2.485) | 4468(3677-5430) | 223.93(183.76-272.76) | 5236(3647-7135) | 267.15(185.23-365.17) | 0.499  (0.373 to 0.625) |
| Djibouti | 6(4-9) | 6.34(4.12-9.37) | 32(20-50) | 7.34(4.61-11.45) | 0.393  (0.17 to 0.617) | 412(299-560) | 404.99(293.7-552.35) | 2432(1743-3350) | 546.99(391.52-755.12) | 0.904  (0.739 to 1.069) |
| Dominica | 2(1-2) | 9.25(7.38-11.34) | 2(2-3) | 10.57(7.64-14.33) | 0.382  (0.187 to 0.577) | 155(124-192) | 824.06(661.97-1020.22) | 286(216-377) | 1245.39(943.18-1642.42) | 1.315  (1.243 to 1.387) |
| Dominican Republic | 102(78-130) | 5.57(4.27-7.11) | 296(212-402) | 7.72(5.52-10.48) | 1.665  (1.411 to 1.919) | 10227(8071-13146) | 550.16(435.27-709.74) | 38878(28676-51297) | 1011.46(745.95-1334.97) | 2.298  (2.184 to 2.412) |
| Ecuador | 110(99-122) | 4.36(3.91-4.82) | 284(214-367) | 4.7(3.54-6.08) | -0.248  (-0.78 to 0.286) | 9558(7917-11656) | 369.79(306.66-450.74) | 35485(26860-45568) | 584.96(442.87-751.13) | 1.227  (0.974 to 1.481) |
| Egypt | 756(592-950) | 5.11(4-6.42) | 1897(1377-2547) | 5.78(4.18-7.77) | 0.52  (0.39 to 0.651) | 53635(43095-65482) | 357.32(286.91-436.34) | 227945(172823-297164) | 685.14(519.58-893.68) | 2.233  (2.144 to 2.322) |
| El Salvador | 78(64-95) | 6.19(5.05-7.49) | 215(160-284) | 10.42(7.74-13.77) | 1.667  (1.438 to 1.897) | 6807(5488-8295) | 529.84(427.72-644.92) | 20047(15520-25402) | 967.95(749.58-1226.71) | 1.898  (1.762 to 2.033) |
| Equatorial Guinea | 10(7-15) | 11.24(7.6-16.59) | 41(24-64) | 10.86(6.25-17.01) | -0.143  (-0.531 to 0.246) | 685(501-946) | 723.48(529.65-1002.55) | 3715(2623-5080) | 935.51(660.29-1280.17) | 0.853  (0.587 to 1.119) |
| Eritrea | 88(59-128) | 11.54(7.72-16.78) | 201(123-316) | 10.69(6.52-16.73) | -0.243  (-0.329 to -0.157) | 5379(3900-7379) | 693.24(503.14-952.02) | 15769(11290-22007) | 814.35(582.58-1139.1) | 0.537  (0.468 to 0.605) |
| Estonia | 11(10-13) | 2.1(1.84-2.37) | 13(11-16) | 2.82(2.36-3.3) | -0.745  (-1.801 to 0.323) | 1308(1027-1652) | 239(187.74-301.76) | 2046(1521-2746) | 424.11(316.96-567.59) | 1.213  (0.873 to 1.553) |
| Eswatini | 13(9-18) | 7.84(5.32-10.97) | 47(26-74) | 15(8.35-23.48) | 2.6  (1.611 to 3.598) | 859(637-1121) | 515.83(381.57-674.39) | 3199(2128-4533) | 975.82(644.54-1390.65) | 2.434  (1.644 to 3.229) |
| Ethiopia | 1614(1292-2094) | 15.02(12.03-19.54) | 1664(1342-2048) | 6.12(4.92-7.54) | -3.535  (-3.793 to -3.278) | 98227(81270-123039) | 891.65(738-1119.09) | 147504(118540-179139) | 524.74(421.74-636) | -2.184  (-2.396 to -1.971) |
| Fiji | 91(70-116) | 42.66(32.56-54.46) | 157(114-213) | 49.83(36.01-67.53) | 0.561  (0.406 to 0.717) | 5364(4209-6645) | 2456.12(1927.41-3041.01) | 10478(8139-13613) | 3309.29(2570.29-4301.04) | 0.989  (0.887 to 1.092) |
| Finland | 38(33-43) | 1.84(1.63-2.07) | 16(14-18) | 0.86(0.75-0.98) | -2.538  (-2.765 to -2.311) | 6528(4832-8535) | 324.19(239.26-424.97) | 9247(6263-13090) | 513.44(347.09-726.93) | 1.383  (1.235 to 1.532) |
| France | 233(209-258) | 1.15(1.04-1.28) | 165(146-185) | 0.73(0.64-0.82) | -1.547  (-1.953 to -1.139) | 28054(21962-35381) | 138.18(108.16-174.33) | 50691(34862-70436) | 234.73(160.92-326.18) | 1.889  (1.782 to 1.997) |
| Gabon | 26(17-38) | 12.04(8.2-17.64) | 65(41-103) | 12.36(7.74-19.49) | -0.118  (-0.296 to 0.06) | 1733(1261-2377) | 786.84(573.9-1078.67) | 5535(4002-7558) | 1030.65(745.42-1406.97) | 0.696  (0.571 to 0.82) |
| Gambia | 10(6-14) | 4.66(3.06-6.73) | 40(26-58) | 6.95(4.55-10.1) | 0.986  (0.725 to 1.247) | 806(594-1058) | 369.1(271.75-485.45) | 3908(2911-5163) | 647.18(482.02-854.1) | 1.661  (1.51 to 1.812) |
| Georgia | 47(41-54) | 2.75(2.42-3.13) | 48(41-56) | 3.71(3.17-4.32) | 1.12  (0.642 to 1.6) | 4915(3912-6273) | 284.12(226.23-362.73) | 7206(5429-9641) | 562.49(423.66-751.65) | 2.222  (1.974 to 2.47) |
| Germany | 554(495-616) | 1.89(1.69-2.11) | 306(269-344) | 1.07(0.94-1.2) | -1.892  (-2.027 to -1.756) | 56157(45327-69613) | 192.64(155.55-238.83) | 88193(61520-119428) | 315.82(219.81-428.25) | 1.602  (1.416 to 1.789) |
| Ghana | 197(137-273) | 5.67(3.95-7.87) | 770(516-1077) | 7.95(5.32-11.11) | 1.546  (1.207 to 1.885) | 15567(12050-20044) | 434.7(335.95-560.61) | 71486(53904-94953) | 712.23(537.33-947.41) | 1.89  (1.689 to 2.092) |
| Greece | 27(24-30) | 0.75(0.67-0.84) | 25(22-29) | 0.64(0.56-0.72) | -0.679  (-0.931 to -0.425) | 7522(5277-10408) | 211.99(148.7-293.17) | 14980(9887-21153) | 408.53(269.12-576.16) | 1.981  (1.9 to 2.061) |
| Greenland | 1(1-1) | 3.56(2.6-4.78) | 0(0-1) | 2.05(1.39-2.84) | -1.918  (-2.036 to -1.799) | 47(36-60) | 214.49(163.97-276.5) | 48(35-62) | 260.36(192.5-339.76) | 0.456  (0.262 to 0.649) |
| Grenada | 3(3-4) | 16.71(14.51-19.09) | 5(4-6) | 12.65(10.18-15.35) | -1.133  (-1.374 to -0.892) | 249(212-294) | 1231.36(1046.43-1454.67) | 495(392-632) | 1354.42(1073.53-1729.06) | 0.147  (0.006 to 0.288) |
| Guam | 2(2-3) | 5.73(4.6-7.11) | 4(3-5) | 6.63(5.29-8.33) | 0.703  (0.43 to 0.978) | 258(201-327) | 584.67(459.54-734.65) | 501(376-657) | 943.32(705.32-1238.35) | 1.564  (1.467 to 1.661) |
| Guatemala | 102(91-113) | 5.52(4.96-6.14) | 874(730-1040) | 18.72(15.61-22.28) | 2.894  (2.472 to 3.319) | 9797(8010-12018) | 525.53(429.05-646) | 72923(60126-89445) | 1536.17(1268.25-1884.47) | 2.992  (2.731 to 3.254) |
| Guinea | 70(47-100) | 5(3.36-7.17) | 196(134-281) | 6.48(4.42-9.26) | 0.879  (0.665 to 1.093) | 5324(3983-6973) | 374.01(280.06-491.1) | 18571(14052-24123) | 585.24(442.97-759.27) | 1.44  (1.367 to 1.514) |
| Guinea-Bissau | 20(14-27) | 9.49(6.58-13.08) | 52(34-75) | 10.54(6.88-15.45) | 0.316  (0.126 to 0.506) | 1395(1057-1816) | 646.79(488.24-844.55) | 4570(3357-6087) | 883.06(645-1178.62) | 0.96  (0.824 to 1.097) |
| Guyana | 37(31-44) | 18.71(15.63-22.23) | 48(34-65) | 18.78(13.4-25.43) | -0.162  (-0.587 to 0.264) | 2987(2460-3656) | 1470.07(1212.1-1798.54) | 5229(3980-6832) | 2065.15(1571.75-2698.27) | 1.039  (0.803 to 1.276) |
| Haiti | 293(209-405) | 18.66(13.36-25.74) | 593(378-894) | 14.46(9.2-21.81) | -0.641  (-0.743 to -0.538) | 21205(16176-27378) | 1325.28(1012.62-1707.81) | 61143(45351-80624) | 1467.78(1087.97-1934.7) | 0.446  (0.373 to 0.519) |
| Honduras | 45(34-58) | 4.44(3.41-5.75) | 132(81-203) | 4.43(2.74-6.81) | -0.351  (-0.584 to -0.117) | 5311(4064-6974) | 511.49(392.08-672.75) | 24014(16960-33488) | 782.88(553.72-1092.33) | 1.279  (1.175 to 1.383) |
| Hungary | 121(108-135) | 3.13(2.8-3.48) | 70(59-81) | 1.77(1.49-2.05) | -1.985  (-2.286 to -1.683) | 12455(9937-15587) | 320.15(256.02-399.75) | 13581(9686-18510) | 335.04(240.46-454.55) | 0.137  (0.015 to 0.259) |
| Iceland | 1(1-1) | 0.71(0.62-0.81) | 1(1-1) | 0.5(0.42-0.59) | -1.236  (-1.415 to -1.057) | 149(105-204) | 170.67(121.09-233.65) | 403(268-570) | 328.14(218.01-463.85) | 2.146  (2.077 to 2.216) |
| India | 10336(9131-11534) | 4.34(3.84-4.85) | 22180(19031-25277) | 4.48(3.85-5.11) | 0.093  (0.03 to 0.156) | 1002667(824004-1215060) | 412.8(339.95-498.83) | 3105893(2400100-3984133) | 620.23(480.02-794.43) | 1.226  (1.128 to 1.323) |
| Indonesia | 2600(2161-3157) | 5.11(4.25-6.2) | 5851(4597-7636) | 5.28(4.15-6.89) | 0.159  (0.077 to 0.242) | 201893(167057-242095) | 388.48(321.75-464.99) | 549031(436179-671822) | 498.21(395.63-609.92) | 0.056  (-0.193 to 0.305) |
| Iran (Islamic Republic of) | 189(159-234) | 1.58(1.33-1.95) | 706(620-790) | 1.95(1.71-2.18) | 1.161  (0.978 to 1.346) | 27360(21219-35371) | 220.36(171.11-284.43) | 158148(116433-210940) | 431.16(317.75-575.02) | 2.13  (2.003 to 2.258) |
| Iraq | 300(216-410) | 7.68(5.54-10.56) | 822(557-1213) | 6.02(4.08-8.87) | -0.908  (-1.022 to -0.795) | 29574(22703-37698) | 725.54(557.17-925.1) | 154409(110653-208167) | 1121.07(804.35-1510.69) | 1.408  (1.335 to 1.481) |
| Ireland | 11(10-12) | 0.96(0.85-1.08) | 10(8-11) | 0.5(0.43-0.58) | -2.128  (-2.469 to -1.786) | 2222(1627-2963) | 191.4(140.12-255.23) | 5191(3479-7301) | 282.54(188.98-397.26) | 1.319  (1.246 to 1.391) |
| Israel | 34(31-38) | 2.29(2.06-2.53) | 43(38-49) | 1.34(1.18-1.51) | -1.795  (-2.338 to -1.249) | 4073(3193-5190) | 268.88(211.25-342.37) | 10024(7147-13646) | 315.1(224.23-429.26) | 0.418  (0.244 to 0.591) |
| Italy | 293(282-304) | 1.43(1.38-1.49) | 149(142-157) | 0.63(0.59-0.66) | -2.769  (-2.938 to -2.599) | 47638(36254-62194) | 236.28(179.56-308.93) | 60676(42483-84537) | 284.36(197.2-399.16) | 0.508  (0.367 to 0.65) |
| Jamaica | 50(45-55) | 8.92(8.02-9.86) | 87(63-120) | 8.7(6.26-11.95) | -0.619  (-1.082 to -0.155) | 3840(3243-4545) | 664.26(562.28-784.95) | 8931(6846-11621) | 888.91(681.24-1157.47) | 0.516  (0.219 to 0.815) |
| Japan | 698(677-718) | 1.36(1.32-1.4) | 236(228-245) | 0.5(0.48-0.52) | -3.258  (-3.589 to -2.926) | 119588(90328-156010) | 238.01(179.43-311.1) | 158453(108782-221404) | 356.46(243.46-499.33) | 1.274  (1.178 to 1.37) |
| Jordan | 48(36-63) | 6.34(4.75-8.28) | 162(118-217) | 3.83(2.78-5.11) | -2.172  (-2.504 to -1.839) | 4452(3493-5651) | 570.78(448.08-724.92) | 33306(23736-44706) | 780.12(556.28-1047.11) | 0.995  (0.908 to 1.082) |
| Kazakhstan | 91(80-102) | 1.89(1.67-2.13) | 121(101-147) | 1.75(1.46-2.13) | -1.865  (-2.604 to -1.121) | 13580(10354-17760) | 279.03(212.18-365.97) | 34967(24583-48272) | 505.07(355.05-697.19) | 1.318  (1.038 to 1.599) |
| Kenya | 189(149-240) | 4.18(3.3-5.31) | 788(622-992) | 5.78(4.56-7.27) | 1.208  (0.863 to 1.555) | 13565(11158-16367) | 288.3(237.53-348.15) | 56688(46673-69245) | 405.5(334.02-494.71) | 1.197  (0.948 to 1.447) |
| Kiribati | 7(5-9) | 37.43(27.3-49.98) | 17(11-25) | 47.5(31.89-70.06) | 0.625  (0.432 to 0.817) | 438(337-560) | 2291.12(1760.81-2934.33) | 1211(893-1619) | 3281.13(2417.97-4383.9) | 1.052  (0.886 to 1.217) |
| Kuwait | 16(14-19) | 2.91(2.53-3.33) | 48(38-60) | 1.87(1.49-2.33) | -1.94  (-2.558 to -1.319) | 2883(2114-3849) | 474.61(349.25-632.68) | 22256(14665-31025) | 862.99(568.54-1205.52) | 1.873  (1.726 to 2.019) |
| Kyrgyzstan | 19(16-22) | 1.77(1.5-2.07) | 46(36-58) | 2.09(1.65-2.61) | -0.312  (-0.741 to 0.119) | 2347(1825-3024) | 215.95(167.29-278.9) | 8522(6229-11483) | 384.93(281.21-519.43) | 1.489  (1.297 to 1.682) |
| Lao People's Democratic Republic | 93(64-133) | 9.71(6.62-13.83) | 168(114-239) | 7.02(4.74-9.97) | -1.311  (-1.424 to -1.198) | 5938(4352-8004) | 606.43(443.96-817.67) | 15236(11328-19957) | 625.74(464.53-819.11) | -0.154  (-0.242 to -0.066) |
| Latvia | 25(23-28) | 2.68(2.41-3) | 27(23-32) | 4.12(3.47-4.75) | -0.178  (-1.39 to 1.05) | 2415(1962-3011) | 258.81(210.48-322.39) | 3190(2538-4107) | 477(380.6-611.55) | 1.25  (0.746 to 1.756) |
| Lebanon | 55(39-75) | 6.47(4.59-8.83) | 71(48-97) | 3.41(2.33-4.67) | -1.999  (-2.254 to -1.743) | 4926(3751-6463) | 580.46(441.93-761.79) | 16883(11981-23288) | 791.51(562.31-1092.72) | 1.269  (1.146 to 1.392) |
| Lesotho | 13(9-18) | 3.68(2.48-5.2) | 54(34-79) | 10.93(6.75-15.81) | 4.58  (3.847 to 5.319) | 991(745-1298) | 272.44(204.9-357.3) | 3825(2718-5224) | 735.9(520.61-1007.32) | 3.966  (3.458 to 4.476) |
| Liberia | 27(19-38) | 5.11(3.51-7.18) | 118(75-181) | 7.32(4.61-11.21) | 1.213  (1.031 to 1.396) | 2260(1694-2947) | 405.47(304.46-529.5) | 11865(8564-16071) | 721.91(521.09-978.55) | 1.962  (1.855 to 2.07) |
| Libya | 21(15-28) | 2.21(1.56-2.99) | 124(79-180) | 3.98(2.54-5.75) | 2.633  (2.38 to 2.887) | 2910(2153-3857) | 302.65(224.09-401.39) | 22309(15907-30252) | 722.45(514.55-980.04) | 3.239  (3.058 to 3.42) |
| Lithuania | 23(21-26) | 1.84(1.63-2.06) | 30(25-35) | 3.19(2.68-3.73) | 0.559  (-0.476 to 1.604) | 2568(2013-3265) | 202.8(158.87-257.94) | 3721(2903-4847) | 396.63(310.26-514.89) | 1.651  (1.284 to 2.018) |
| Luxembourg | 2(2-2) | 1.4(1.25-1.57) | 1(1-1) | 0.43(0.36-0.5) | -3.951  (-4.205 to -3.697) | 311(234-408) | 211.31(159.26-276.91) | 802(529-1148) | 312.98(205.93-448.41) | 1.197  (1.12 to 1.275) |
| Madagascar | 183(134-247) | 7.25(5.31-9.78) | 491(331-708) | 6.65(4.48-9.59) | -0.255  (-0.328 to -0.182) | 12018(9349-15467) | 459.23(356.61-591.25) | 38587(28811-50328) | 511.31(381.37-667.75) | 0.348  (0.28 to 0.417) |
| Malawi | 164(119-219) | 7.88(5.75-10.54) | 410(288-571) | 8.88(6.25-12.37) | 0.056  (-0.262 to 0.375) | 10009(7660-12833) | 467.01(357.24-598.7) | 26595(20074-35029) | 559.26(421.66-737.56) | 0.29  (0.03 to 0.551) |
| Malaysia | 221(170-284) | 4.59(3.53-5.89) | 466(361-587) | 4.08(3.16-5.14) | -0.465  (-0.717 to -0.212) | 20745(16490-26135) | 416.5(332.01-523.94) | 60344(45791-78976) | 518.84(394.21-678.66) | 0.669  (0.565 to 0.772) |
| Maldives | 4(3-5) | 8.16(5.81-11.08) | 6(4-9) | 2.61(1.81-3.63) | -3.943  (-4.249 to -3.636) | 238(181-308) | 529.67(401.44-685.86) | 935(693-1249) | 368.74(273.07-493.5) | -1.343  (-1.641 to -1.045) |
| Mali | 115(83-159) | 6.04(4.37-8.35) | 312(218-440) | 6.33(4.43-8.93) | 0.117  (0.032 to 0.203) | 10791(8243-14007) | 549.29(419.8-713.54) | 43629(32323-58312) | 841.55(625.64-1123.15) | 1.496  (1.366 to 1.625) |
| Malta | 3(2-3) | 1.97(1.75-2.23) | 2(2-3) | 1.45(1.25-1.68) | -0.985  (-1.168 to -0.801) | 332(262-426) | 232.57(183.03-298) | 744(516-1022) | 462.88(319.53-636.59) | 2.222  (2.078 to 2.365) |
| Marshall Islands | 2(2-3) | 23.93(16.97-33.17) | 9(5-13) | 44.31(26.46-69.66) | 1.862  (1.457 to 2.268) | 178(135-229) | 1868(1418.51-2403.27) | 754(542-1018) | 3857.04(2772.66-5207.12) | 2.317  (2.041 to 2.593) |
| Mauritania | 21(15-29) | 4.77(3.36-6.44) | 48(32-72) | 4.62(3.05-6.83) | -0.395  (-0.504 to -0.285) | 1626(1220-2073) | 350.77(262.63-447.25) | 4564(3379-6054) | 421.63(311.91-559.55) | 0.34  (0.272 to 0.408) |
| Mauritius | 51(45-56) | 15.24(13.67-16.9) | 124(109-138) | 24.94(22-27.8) | 2.988  (2.384 to 3.596) | 3350(2936-3832) | 982.06(861.87-1122.56) | 8604(7482-10120) | 1753.68(1524.26-2063.1) | 2.863  (2.467 to 3.261) |
| Mexico | 2846(2747-2944) | 13.6(13.13-14.07) | 7058(6229-7992) | 14.68(12.96-16.61) | -0.057  (-0.466 to 0.354) | 249603(210536-294497) | 1161.78(981.49-1367.61) | 654443(540124-790368) | 1371.25(1130.93-1657.2) | 0.388  (0.239 to 0.538) |
| Micronesia (Federated States of) | 5(3-7) | 22.08(14.34-32.02) | 10(6-14) | 29.24(19.66-42.82) | 0.858  (0.577 to 1.139) | 347(252-469) | 1487.3(1073.13-2013.99) | 779(579-1023) | 2369.62(1760.77-3111.03) | 1.574  (1.33 to 1.819) |
| Monaco | 0(0-0) | 0.52(0.36-0.72) | 0(0-0) | 0.57(0.35-0.86) | 0.322  (0.202 to 0.442) | 18(12-25) | 156.04(109.37-217.29) | 36(24-50) | 313(208.81-441.86) | 2.277  (2.249 to 2.305) |
| Mongolia | 10(7-13) | 1.99(1.44-2.65) | 34(25-45) | 2.78(2.01-3.67) | 0.976  (0.702 to 1.251) | 1085(823-1384) | 213.98(162.72-272.79) | 5342(3996-7083) | 433.18(323.99-574.21) | 2.265  (2.169 to 2.361) |
| Montenegro | 6(4-7) | 2.82(2.18-3.56) | 6(4-7) | 2.47(1.86-3.2) | -0.405  (-0.682 to -0.128) | 645(497-833) | 314.04(241.71-406.05) | 931(676-1262) | 400.21(291.17-541.4) | 0.769  (0.637 to 0.901) |
| Morocco | 129(92-177) | 2.02(1.44-2.78) | 403(255-616) | 2.99(1.89-4.57) | 1.513  (1.33 to 1.696) | 22151(16159-29991) | 337.24(246.23-456.52) | 116795(79518-162987) | 868.4(591.1-1212.04) | 3.245  (3.161 to 3.329) |
| Mozambique | 243(173-335) | 7.8(5.58-10.76) | 823(524-1208) | 11.95(7.61-17.5) | 2.102  (1.857 to 2.347) | 14841(11295-19331) | 469.47(357.54-612.11) | 56272(40282-77172) | 794.92(568.75-1090.87) | 2.288  (2.097 to 2.479) |
| Myanmar | 1881(1280-2678) | 18.01(12.22-25.68) | 2371(1655-3298) | 12.11(8.45-16.84) | -1.663  (-1.817 to -1.509) | 115194(83687-155242) | 1077.77(780.34-1453.98) | 190155(145756-245318) | 973.22(746.1-1255.56) | -0.7  (-0.827 to -0.573) |
| Namibia | 15(11-21) | 5.14(3.61-7.12) | 45(27-67) | 6.25(3.81-9.38) | 0.173  (-0.377 to 0.725) | 1136(869-1483) | 366.57(280.47-478.92) | 3716(2660-5078) | 501.96(359.05-686.6) | 0.686  (0.305 to 1.068) |
| Nauru | 1(1-1) | 31.95(21.91-44.83) | 1(1-2) | 39.86(26.7-57.32) | 0.529  (0.321 to 0.737) | 57(43-76) | 2048.62(1519.66-2722.77) | 100(74-131) | 3024.6(2244.96-3979.96) | 1.055  (0.878 to 1.233) |
| Nepal | 207(144-288) | 4.27(2.97-5.94) | 434(292-638) | 4.54(3.05-6.68) | 0.223  (-0.07 to 0.516) | 22101(16821-28861) | 446.38(339.88-582.25) | 72843(53300-97777) | 744.97(545.76-998.9) | 1.635  (1.375 to 1.895) |
| Netherlands | 113(101-125) | 1.96(1.75-2.18) | 44(39-49) | 0.75(0.67-0.84) | -3.18  (-3.481 to -2.879) | 12401(9877-15468) | 215.59(171.59-269.07) | 16408(11080-22918) | 292.11(196.95-408.01) | 0.936  (0.829 to 1.044) |
| New Zealand | 26(23-29) | 2.17(1.96-2.39) | 25(22-27) | 1.37(1.24-1.5) | -2.172  (-2.604 to -1.738) | 2832(2279-3551) | 235.43(189.56-295.05) | 5217(3928-6851) | 291.26(219.22-382.61) | 0.635  (0.361 to 0.909) |
| Nicaragua | 46(36-57) | 5.77(4.52-7.15) | 127(95-168) | 5.8(4.3-7.68) | -0.006  (-0.19 to 0.178) | 4682(3654-5929) | 569.86(445.08-721.31) | 17816(13325-23250) | 799.03(597.84-1041.98) | 1.028  (0.953 to 1.104) |
| Niger | 63(43-90) | 3.7(2.51-5.32) | 171(110-259) | 3.79(2.44-5.75) | -0.054  (-0.197 to 0.089) | 5742(4290-7593) | 327.58(244.47-433.13) | 22948(16735-31024) | 479.47(350.72-647.77) | 1.15  (1.078 to 1.222) |
| Nigeria | 1180(909-1522) | 5.62(4.33-7.24) | 2794(1914-3860) | 5.13(3.52-7.08) | -0.518  (-0.625 to -0.41) | 84688(68427-104017) | 393.49(318.22-483.29) | 254210(196704-328298) | 454.81(352.67-586.38) | 0.323  (0.248 to 0.399) |
| Niue | 0(0-0) | 16.7(10.89-24.67) | 0(0-0) | 23.18(15.2-34.78) | 0.687  (0.492 to 0.883) | 9(6-12) | 1349.7(987.92-1800.11) | 14(10-19) | 2469.39(1822.94-3266.07) | 1.776  (1.641 to 1.911) |
| North Macedonia | 27(22-34) | 3.98(3.18-4.9) | 25(18-34) | 2.79(2.01-3.74) | -1.163  (-1.435 to -0.89) | 2458(1975-3072) | 357.56(287.28-447.14) | 3787(2787-5105) | 412.64(304.23-555.06) | 0.422  (0.285 to 0.56) |
| Northern Mariana Islands | 2(1-3) | 13.26(8.82-19.32) | 2(2-3) | 11.26(8.38-14.85) | -0.519  (-0.886 to -0.151) | 170(126-226) | 961.6(717.03-1281.66) | 234(180-305) | 1228.13(939.41-1608.61) | 0.749  (0.561 to 0.936) |
| Norway | 27(26-28) | 1.74(1.66-1.83) | 19(18-20) | 0.98(0.92-1.03) | -2.136  (-2.676 to -1.594) | 4684(3554-6119) | 303.6(230.3-396.72) | 6629(4709-9065) | 345.07(244.69-472.4) | 0.11  (-0.041 to 0.262) |
| Oman | 27(18-42) | 5.09(3.3-7.82) | 87(62-120) | 4.2(2.94-5.76) | -0.274  (-0.601 to 0.054) | 2500(1853-3302) | 446.53(328.47-592.52) | 13467(9999-17790) | 617.95(459.52-816.92) | 0.784  (0.702 to 0.865) |
| Pakistan | 1328(1022-1714) | 5.12(3.94-6.61) | 5081(3730-6831) | 7.3(5.37-9.81) | 0.752  (0.442 to 1.062) | 128340(100728-158728) | 488.88(384.27-604.1) | 613848(465454-787373) | 865(657.33-1108.75) | 1.757  (1.551 to 1.963) |
| Palau | 1(1-1) | 18.66(12.26-26.99) | 2(1-3) | 24.41(16.85-34.23) | 0.909  (0.716 to 1.103) | 71(52-93) | 1440.69(1049.96-1894.21) | 193(146-254) | 2434.38(1836.11-3187.33) | 1.769  (1.66 to 1.878) |
| Palestine | 18(12-26) | 4.66(3.11-6.67) | 56(42-74) | 3.93(2.91-5.16) | -0.628  (-0.697 to -0.559) | 1628(1222-2144) | 411.43(308.36-542.41) | 8761(6473-11436) | 599.36(443.35-782.08) | 1.166  (1.104 to 1.228) |
| Panama | 28(25-31) | 4.15(3.7-4.68) | 69(54-86) | 4.75(3.69-5.92) | 0.328  (0.068 to 0.59) | 3016(2388-3835) | 442.58(350.89-562.9) | 10144(7574-13452) | 696.45(520.07-923.34) | 1.44  (1.325 to 1.555) |
| Papua New Guinea | 236(144-344) | 23.32(14.27-33.94) | 689(490-945) | 21.88(15.58-30.05) | -0.252  (-0.311 to -0.193) | 15664(11016-21171) | 1502.81(1059.65-2035.03) | 60774(46660-78423) | 1896.32(1456.76-2445.45) | 0.686  (0.637 to 0.736) |
| Paraguay | 47(37-58) | 4.58(3.64-5.66) | 173(124-234) | 7.33(5.25-9.96) | 1.778  (1.506 to 2.05) | 4283(3435-5475) | 414.17(331.65-530.1) | 16601(12844-21467) | 695.64(537.75-900.49) | 1.852  (1.674 to 2.03) |
| Peru | 153(116-197) | 2.77(2.11-3.57) | 400(278-564) | 3.11(2.16-4.38) | 0.28  (-0.017 to 0.577) | 13429(10529-16927) | 237.73(186.57-299.39) | 41703(31455-54898) | 322.52(243.28-424.59) | 0.981  (0.747 to 1.216) |
| Philippines | 1269(1146-1410) | 7.82(7.06-8.68) | 3363(2820-3955) | 9.08(7.61-10.69) | 0.447  (0.297 to 0.597) | 91632(80191-105303) | 549.96(482.03-630.74) | 238129(202456-280321) | 637.97(542.53-750.72) | 0.399  (0.235 to 0.564) |
| Poland | 465(446-485) | 3.51(3.37-3.66) | 356(321-390) | 2.28(2.06-2.5) | -0.973  (-1.481 to -0.463) | 46034(38136-55840) | 346.43(286.84-420.86) | 59597(45135-77459) | 377.42(286.52-489.94) | 0.424  (0.161 to 0.687) |
| Portugal | 89(80-99) | 2.63(2.36-2.93) | 41(36-46) | 0.99(0.87-1.12) | -3.466  (-3.844 to -3.087) | 10870(8434-13756) | 321.63(249.61-407) | 17921(12149-25027) | 466.33(314.58-652.41) | 1.026  (0.916 to 1.136) |
| Puerto Rico | 122(110-135) | 10.08(9.07-11.13) | 107(86-132) | 9.24(7.44-11.35) | -0.81  (-1.115 to -0.504) | 10605(8852-12793) | 873.75(729.37-1053.99) | 13433(10308-17430) | 1177.01(903.37-1525.29) | 0.775  (0.656 to 0.894) |
| Qatar | 8(6-12) | 4.55(3.08-6.54) | 60(41-86) | 3.68(2.49-5.27) | -0.887  (-1.331 to -0.44) | 847(625-1101) | 447.64(331.92-580.42) | 12753(8970-17379) | 746.47(526.19-1020.98) | 1.411  (1.201 to 1.621) |
| Republic of Korea | 803(656-976) | 5.36(4.38-6.52) | 310(248-392) | 1.41(1.12-1.78) | -4.845  (-5.186 to -4.502) | 74263(59263-92162) | 485.78(387.99-602.41) | 142577(96877-201157) | 685.22(465.18-965.96) | 1.066  (0.827 to 1.305) |
| Republic of Moldova | 30(27-34) | 2.04(1.82-2.28) | 47(41-55) | 3.16(2.73-3.66) | -0.21  (-1.3 to 0.892) | 3793(2934-4937) | 257.66(198.89-335.99) | 7277(5463-9563) | 480.21(361.72-629.56) | 1.444  (1.144 to 1.744) |
| Romania | 163(145-182) | 2.14(1.91-2.39) | 81(69-95) | 1.09(0.92-1.28) | -2.504  (-2.789 to -2.219) | 17647(14009-22577) | 230.63(183.33-294.79) | 18430(12971-25638) | 243.45(172.24-337.36) | 0.04  (-0.071 to 0.152) |
| Russian Federation | 728(711-746) | 1.42(1.39-1.45) | 1298(1188-1400) | 2.3(2.1-2.48) | -0.241  (-1.021 to 0.544) | 97470(77179-122825) | 190.6(150.54-240.56) | 196958(153067-252357) | 344.29(268.23-440.46) | 1.188  (0.901 to 1.475) |
| Rwanda | 190(123-286) | 13.18(8.59-19.75) | 252(136-414) | 6.9(3.72-11.3) | -3.443  (-3.947 to -2.936) | 11154(7735-15778) | 743.03(516.82-1048.99) | 17916(11746-26516) | 476.57(311.42-706.66) | -2.622  (-3.068 to -2.174) |
| Saint Kitts and Nevis | 1(1-2) | 14.56(12.85-16.46) | 1(1-2) | 5.67(4.2-7.64) | -3.585  (-4.071 to -3.097) | 108(92-128) | 1106.05(944.95-1315.68) | 220(164-296) | 896.92(670.1-1208.54) | -0.964  (-1.267 to -0.659) |
| Saint Lucia | 6(5-6) | 17.99(16.1-20.1) | 8(6-10) | 10.7(8.54-13.24) | -1.823  (-1.997 to -1.648) | 443(376-526) | 1345.55(1142.91-1599.97) | 956(737-1246) | 1335.07(1031.35-1738.46) | -0.089  (-0.203 to 0.025) |
| Saint Vincent and the Grenadines | 5(4-5) | 19.47(17.21-22.06) | 7(6-8) | 15.79(13.18-18.87) | -1.054  (-1.215 to -0.892) | 349(300-408) | 1376.13(1182.01-1609.33) | 651(520-820) | 1574.24(1255.19-1982.16) | 0.152  (0.044 to 0.26) |
| Samoa | 5(4-7) | 14.64(10.14-20.71) | 12(8-17) | 20.13(13.48-29.31) | 0.997  (0.873 to 1.121) | 433(323-570) | 1195.06(891.41-1568.61) | 1258(928-1653) | 2125.22(1570.4-2791.24) | 1.927  (1.885 to 1.969) |
| San Marino | 0(0-0) | 0.76(0.56-1.01) | 0(0-0) | 0.4(0.21-0.65) | -1.275  (-1.59 to -0.959) | 15(11-20) | 174.91(123.76-239.15) | 35(23-50) | 311.65(204.15-445.14) | 1.936  (1.891 to 1.981) |
| Sao Tome and Principe | 0(0-1) | 1.99(1.41-2.69) | 2(1-2) | 2.51(1.59-3.8) | 0.444  (0.099 to 0.79) | 62(46-82) | 261.6(194.98-347.58) | 330(233-454) | 497.63(351.97-684) | 1.986  (1.881 to 2.092) |
| Saudi Arabia | 154(101-226) | 3.76(2.45-5.54) | 1152(768-1651) | 5.98(4-8.56) | 1.716  (1.577 to 1.855) | 17663(13074-23223) | 414.22(306.86-544.46) | 162861(119049-215535) | 835.28(610.16-1104.28) | 2.457  (2.351 to 2.562) |
| Senegal | 87(62-120) | 5.55(3.94-7.67) | 258(172-375) | 6.56(4.38-9.56) | 0.608  (0.404 to 0.813) | 7804(6058-10070) | 479.53(372.53-618.88) | 28733(21436-37548) | 702.16(524.11-917.5) | 1.405  (1.266 to 1.544) |
| Serbia | 119(93-149) | 3.54(2.77-4.42) | 82(62-106) | 2.39(1.8-3.08) | -1.597  (-1.822 to -1.371) | 12189(9405-15623) | 360.56(278.23-461.91) | 13607(9854-18606) | 392.18(284.71-534.59) | 0.062  (-0.04 to 0.165) |
| Seychelles | 1(1-1) | 3.77(2.99-4.67) | 2(2-3) | 4.71(3.63-5.95) | 1.008  (0.868 to 1.149) | 67(53-85) | 349.58(277.07-441.85) | 326(240-433) | 763.15(562.64-1013.37) | 2.61  (2.521 to 2.699) |
| Sierra Leone | 37(24-52) | 3.87(2.59-5.48) | 117(77-173) | 5.26(3.5-7.77) | 1.272  (1.031 to 1.513) | 3301(2492-4348) | 331.69(250.82-437.14) | 13320(9939-17991) | 571.85(427.82-771.54) | 1.926  (1.787 to 2.066) |
| Singapore | 29(26-32) | 2.42(2.17-2.7) | 9(8-10) | 0.33(0.29-0.37) | -6.623  (-7.24 to -6.002) | 4987(3694-6590) | 414.7(307.21-547.81) | 14018(9129-20515) | 520.68(339.25-762.26) | 0.513  (0.322 to 0.705) |
| Slovakia | 44(35-54) | 2.38(1.9-2.95) | 31(23-40) | 1.37(1.02-1.77) | -1.48  (-1.668 to -1.292) | 4609(3550-5960) | 250.17(192.45-323.73) | 6263(4476-8649) | 270.35(194.02-371.67) | 0.331  (0.261 to 0.401) |
| Slovenia | 16(14-18) | 2.19(1.95-2.47) | 9(7-10) | 1.12(0.91-1.34) | -2.577  (-2.762 to -2.392) | 1730(1368-2204) | 238.1(188.26-303.29) | 2042(1441-2853) | 255.01(181.17-354.27) | 0.049  (-0.027 to 0.125) |
| Solomon Islands | 18(9-29) | 26.18(13.35-41.74) | 81(55-114) | 39.54(26.86-55.94) | 1.379  (1.298 to 1.461) | 1114(668-1656) | 1548.95(922.37-2311.11) | 5203(3844-6880) | 2529.78(1868.88-3346.79) | 1.62  (1.561 to 1.678) |
| Somalia | 195(124-290) | 10.81(6.87-16.08) | 516(326-780) | 10.55(6.66-15.95) | -0.207  (-0.311 to -0.104) | 11785(8181-16478) | 635.72(439.71-891.33) | 36427(25985-49583) | 725.14(515.23-988.1) | 0.32  (0.247 to 0.392) |
| South Africa | 866(769-998) | 8.92(7.92-10.26) | 1797(1554-2045) | 8.83(7.64-10.04) | 0.419  (0.09 to 0.75) | 61697(53477-71300) | 616.86(535.58-710.95) | 145067(120398-171844) | 698.14(580.52-825.22) | 0.644  (0.413 to 0.875) |
| South Sudan | 96(63-141) | 7.85(5.17-11.58) | 239(155-355) | 9.89(6.4-14.72) | 0.624  (0.334 to 0.916) | 5994(4258-8278) | 474.19(335.55-657.6) | 15934(11548-21792) | 659.02(477.42-901.64) | 0.978  (0.753 to 1.204) |
| Spain | 199(178-221) | 1.54(1.38-1.71) | 88(77-99) | 0.48(0.42-0.54) | -4.065  (-4.263 to -3.867) | 33834(25130-45122) | 262.18(194.77-349.58) | 72633(48041-102611) | 416.36(275-588.15) | 1.377  (1.199 to 1.555) |
| Sri Lanka | 328(250-424) | 6.09(4.65-7.88) | 544(321-803) | 6.73(3.99-9.91) | 0.741  (0.483 to 1.001) | 26729(21007-33152) | 488.15(384.14-605.05) | 66626(48316-90007) | 830.14(602.82-1121.22) | 1.985  (1.734 to 2.237) |
| Sudan | 109(72-170) | 2.4(1.59-3.74) | 342(201-535) | 2.91(1.72-4.55) | 0.825  (0.677 to 0.973) | 12471(9360-16555) | 267.17(200.81-354.21) | 63088(45349-86060) | 518.82(373.39-707.39) | 2.26  (2.205 to 2.316) |
| Suriname | 9(7-11) | 8.57(6.39-10.83) | 20(14-27) | 9.67(6.86-13.13) | 0.149  (-0.104 to 0.403) | 838(644-1054) | 783.94(603.02-986.75) | 2639(1965-3538) | 1300.51(969.01-1742.05) | 1.638  (1.521 to 1.755) |
| Sweden | 77(70-84) | 2.36(2.14-2.58) | 38(33-44) | 1.09(0.94-1.26) | -2.032  (-2.206 to -1.859) | 9541(7521-12121) | 297.28(234.15-378.18) | 11863(8403-16186) | 339.69(240.31-463.94) | 0.443  (0.336 to 0.55) |
| Switzerland | 41(37-46) | 1.51(1.35-1.68) | 16(14-18) | 0.48(0.42-0.55) | -3.651  (-3.847 to -3.455) | 7380(5393-9842) | 271.33(198.17-361.74) | 13656(8970-19519) | 420.94(276.07-601.21) | 1.345  (1.175 to 1.516) |
| Syrian Arab Republic | 85(61-114) | 3.26(2.34-4.38) | 119(80-177) | 2.31(1.55-3.41) | -1.488  (-1.875 to -1.1) | 9976(7612-12914) | 374.4(285.49-484.72) | 26497(18626-36139) | 540.99(380.14-738.78) | 1.042  (0.908 to 1.177) |
| Taiwan (Province of China) | 310(281-342) | 4.78(4.33-5.27) | 365(318-410) | 3.61(3.15-4.06) | -1.582  (-2.02 to -1.141) | 29389(23957-36104) | 437(357.22-536.42) | 49822(37247-64840) | 507.28(378.88-660.1) | -0.045  (-0.283 to 0.192) |
| Tajikistan | 42(33-53) | 3.87(3.04-4.85) | 107(75-146) | 3.45(2.42-4.7) | -1.426  (-1.858 to -0.992) | 3519(2781-4392) | 313.03(248.8-389.64) | 13626(10288-18045) | 437.05(329.83-579.28) | 0.352  (0.07 to 0.635) |
| Thailand | 883(631-1181) | 5.08(3.63-6.79) | 1707(1176-2355) | 6.19(4.29-8.51) | -0.197  (-0.691 to 0.299) | 66874(51691-85063) | 375.13(289.9-477.54) | 152666(114809-197701) | 565.3(427.04-730.59) | 0.772  (0.418 to 1.128) |
| Timor-Leste | 8(5-11) | 3.79(2.47-5.57) | 13(9-19) | 3.69(2.48-5.36) | -0.108  (-0.565 to 0.35) | 571(416-770) | 276.46(201.24-372.54) | 1586(1159-2110) | 445.53(325.42-592.73) | 1.568  (1.279 to 1.858) |
| Togo | 34(24-46) | 4.48(3.18-6.18) | 137(87-206) | 5.82(3.71-8.8) | 0.835  (0.576 to 1.094) | 2589(1997-3357) | 331(255.22-429.14) | 11829(8612-15721) | 493.04(358.55-655.97) | 1.246  (1.103 to 1.388) |
| Tokelau | 0(0-0) | 15.53(9.91-22.99) | 0(0-0) | 18.42(12.97-26.32) | 0.31  (0.195 to 0.425) | 5(4-7) | 1354.89(992.2-1854.11) | 10(7-13) | 2176.85(1610.75-2862.22) | 1.413  (1.327 to 1.499) |
| Tonga | 4(3-5) | 17.97(13.54-23.74) | 6(4-9) | 21.44(14.75-30.56) | 0.355  (0.215 to 0.495) | 285(222-355) | 1297.3(1013.71-1614.46) | 580(437-767) | 1963.62(1479.18-2598.25) | 1.22  (1.134 to 1.305) |
| Trinidad and Tobago | 89(81-98) | 25.65(23.32-28.13) | 101(74-132) | 18.83(13.76-24.6) | -1.495  (-1.773 to -1.216) | 5971(5240-6859) | 1682.98(1478.18-1933.95) | 9571(7539-12394) | 1782.42(1405.25-2305.98) | -0.169  (-0.336 to -0.002) |
| Tunisia | 28(21-38) | 1.37(1.02-1.82) | 95(62-138) | 2.04(1.32-2.94) | 1.255  (1.173 to 1.336) | 5206(3767-6997) | 244.52(177.32-328.16) | 26959(18773-38075) | 581.15(404.31-820.71) | 2.76  (2.686 to 2.833) |
| Turkey | 665(493-887) | 4.31(3.2-5.76) | 680(491-921) | 2.04(1.48-2.77) | -2.428  (-2.715 to -2.14) | 54321(42061-69301) | 343.49(266.21-438.34) | 147065(104463-200886) | 448.85(318.69-612.87) | 0.929  (0.588 to 1.271) |
| Turkmenistan | 27(24-30) | 3.29(2.97-3.66) | 120(91-159) | 6.78(5.13-9.04) | 1.822  (1.315 to 2.331) | 2327(1934-2805) | 273.7(228.12-329.21) | 10978(8709-13857) | 620.69(492.29-783.82) | 2.336  (2.039 to 2.635) |
| Tuvalu | 1(0-1) | 20.66(13.88-29.85) | 1(1-1) | 22.84(15.76-31.7) | 0.339  (0.253 to 0.425) | 36(27-48) | 1329.8(969.99-1772.87) | 71(53-91) | 1861.17(1405.37-2410.24) | 1.063  (1.001 to 1.126) |
| Uganda | 209(126-357) | 6.41(3.86-10.94) | 709(439-1130) | 7.66(4.73-12.22) | -0.198  (-0.561 to 0.167) | 13655(9236-21055) | 402.86(272.23-625.17) | 53001(37656-75047) | 552.73(392.01-783.72) | 0.403  (0.122 to 0.684) |
| Ukraine | 383(347-422) | 2.16(1.96-2.38) | 278(193-372) | 1.65(1.15-2.2) | -2.972  (-3.73 to -2.208) | 42838(34680-52510) | 241.69(195.62-296.31) | 58005(41896-77301) | 334.1(241.88-444.45) | -0.038  (-0.408 to 0.333) |
| United Arab Emirates | 22(14-35) | 3.28(2.1-5.22) | 131(82-192) | 1.9(1.21-2.76) | -1.412  (-1.921 to -0.901) | 2370(1715-3182) | 326.07(235.83-441.85) | 33958(23470-46866) | 473.38(328.56-652.2) | 1.195  (1.018 to 1.373) |
| United Kingdom | 295(289-301) | 1.45(1.42-1.48) | 223(216-230) | 0.95(0.92-0.98) | -1.23  (-1.446 to -1.014) | 47979(36171-62577) | 236.54(178.33-308.69) | 128558(87006-178953) | 559.3(377.59-780) | 2.613  (2.481 to 2.744) |
| United Republic of Tanzania | 372(264-507) | 7.05(5-9.65) | 990(650-1416) | 6.67(4.38-9.56) | -0.372  (-0.491 to -0.252) | 23209(17578-30180) | 426.56(322.07-556.54) | 73790(54375-98045) | 487.46(359.09-647.91) | 0.264  (0.188 to 0.341) |
| United States of America | 3625(3519-3734) | 3.87(3.76-3.99) | 4259(4090-4417) | 3.77(3.62-3.91) | -0.357  (-0.598 to -0.115) | 333(260-428) | 342.93(290.82-408.7) | 352(256-476) | 571.36(445.68-729.64) | 1.574  (1.469 to 1.68) |
| United States Virgin Islands | 3(2-4) | 8.15(6.08-10.64) | 2(1-3) | 8(5.2-11.35) | 0.348  (0.199 to 0.498) | 321879(273212-383379) | 827.14(645.79-1061.15) | 643892(502000-822251) | 1262.91(919.22-1704.8) | 1.613  (1.526 to 1.7) |
| Uruguay | 21(19-24) | 2.1(1.87-2.36) | 24(21-27) | 1.98(1.75-2.22) | -0.65  (-0.863 to -0.436) | 1956(1578-2418) | 195.85(158-242.09) | 4016(2975-5397) | 330.93(245.18-444.5) | 1.631  (1.535 to 1.726) |
| Uzbekistan | 149(130-167) | 3.27(2.85-3.7) | 599(479-727) | 4.9(3.91-5.96) | -0.011  (-0.567 to 0.547) | 14220(11624-17544) | 298.99(244.15-369.7) | 73500(56980-95806) | 598.36(463.56-780.77) | 1.51  (1.189 to 1.832) |
| Vanuatu | 6(4-10) | 16.28(10.03-27.24) | 20(13-29) | 21.77(14.83-31.76) | 0.724  (0.641 to 0.808) | 399(278-584) | 1097.67(763.69-1616.57) | 1700(1280-2212) | 1843.48(1389.53-2399.56) | 1.512  (1.458 to 1.565) |
| Venezuela (Bolivarian Republic of) | 304(274-335) | 6.21(5.59-6.84) | 724(533-967) | 7.06(5.2-9.42) | 0.038  (-0.408 to 0.486) | 27784(22917-33758) | 547.08(452.28-663.64) | 80477(61951-104770) | 794.24(611.42-1032.38) | 1.102  (0.848 to 1.357) |
| Viet Nam | 749(510-1047) | 5(3.4-7.01) | 1834(1261-2595) | 4.67(3.21-6.6) | -0.025  (-0.195 to 0.146) | 54932(41028-71657) | 351.05(261.57-457.59) | 163027(124027-215381) | 415.34(315.97-548.72) | 0.57  (0.392 to 0.748) |
| Yemen | 54(32-91) | 2.06(1.23-3.47) | 178(103-310) | 2.04(1.19-3.55) | -0.055  (-0.18 to 0.07) | 6463(4607-8999) | 236.18(168.76-328.64) | 36554(25247-50439) | 395.78(274.44-546.36) | 0.937  (0.548 to 1.328) |
| Zambia | 150(110-203) | 9.56(7-12.93) | 457(296-676) | 9.38(6.07-13.86) | -0.516  (-0.749 to -0.283) | 10165(7877-13013) | 627.14(485.19-803.56) | 39467(29158-52578) | 780.3(575.95-1039.92) | 0.347  (0.192 to 0.502) |
| Zimbabwe | 53(38-70) | 2.69(1.95-3.55) | 270(175-396) | 6.66(4.32-9.77) | 3.721  (2.691 to 4.762) | 5089(3874-6560) | 243.27(185.87-312.52) | 22462(16298-29582) | 535.03(387.5-705.15) | 3.016  (2.378 to 3.659) |

**EAPC: estimated annual percentage changes ;**

**ASR: age-standardized rate;**

**DALYs: disability-adjusted life years.**

**Supplementary Table 3.** Forecast of age-standardized mortality and DALY rates for diabetes mellitus due to metabolic factors based on disease burden from 1990 to 2021, grouped by sex, projected to 2040

| **Year** | **ASMR_per 100,000 (95% CI)** | | | **ASDR_per 100,000 (95% CI)** | | |
| --- | --- | --- | --- | --- | --- | --- |
|  | Both | Male | Female | Both | Male | Female |
| 1990 | 3.83(3.71-3.95) | 4.12(4.08 - 4.16) | 3.52(3.49 - 3.56) | 367.76(366.48-369.04) | 392.61(392.18 - 393.05) | 341.96(341.55 - 342.37) |
| 1991 | 3.85(3.74-3.97) | 4.17(4.13 - 4.21) | 3.53(3.49 - 3.56) | 371.01(369.74-372.27) | 396.9(396.47 - 397.33) | 344.13(343.72 - 344.53) |
| 1992 | 3.89(3.77-4) | 4.21(4.17 - 4.25) | 3.55(3.51 - 3.59) | 375.37(374.11-376.63) | 401.49(401.06 - 401.91) | 348.26(347.86 - 348.66) |
| 1993 | 3.92(3.8-4.03) | 4.25(4.21 - 4.29) | 3.57(3.53 - 3.61) | 379.98(378.73-381.23) | 406.69(406.27 - 407.11) | 352.28(351.88 - 352.68) |
| 1994 | 3.94(3.82-4.05) | 4.28(4.25 - 4.32) | 3.58(3.54 - 3.61) | 384.09(382.85-385.32) | 411.48(411.06 - 411.9) | 355.7(355.3 - 356.09) |
| 1995 | 3.95(3.84-4.06) | 4.31(4.28 - 4.35) | 3.58(3.54 - 3.61) | 387.6(386.38-388.83) | 415.73(415.32 - 416.14) | 358.48(358.09 - 358.87) |
| 1996 | 3.93(3.82-4.04) | 4.3(4.26 - 4.34) | 3.55(3.52 - 3.59) | 391.02(389.8-392.23) | 419.62(419.21 - 420.03) | 361.43(361.05 - 361.82) |
| 1997 | 3.91(3.8-4.02) | 4.28(4.24 - 4.32) | 3.53(3.49 - 3.56) | 396.98(395.77-398.19) | 426.24(425.83 - 426.65) | 366.76(366.38 - 367.15) |
| 1998 | 3.89(3.79-4) | 4.28(4.24 - 4.31) | 3.5(3.47 - 3.53) | 404.71(403.5-405.92) | 435.53(435.12 - 435.94) | 372.93(372.55 - 373.32) |
| 1999 | 3.92(3.81-4.03) | 4.32(4.28 - 4.35) | 3.51(3.48 - 3.54) | 414.78(413.56-415.99) | 447.11(446.7 - 447.52) | 381.5(381.12 - 381.89) |
| 2000 | 3.92(3.82-4.03) | 4.33(4.3 - 4.37) | 3.5(3.46 - 3.53) | 420.86(419.65-422.08) | 454.71(454.3 - 455.12) | 386.07(385.68 - 386.45) |
| 2001 | 3.91(3.8-4.01) | 4.33(4.29 - 4.36) | 3.47(3.44 - 3.51) | 424.34(423.13-425.55) | 458.97(458.56 - 459.38) | 388.77(388.39 - 389.15) |
| 2002 | 3.92(3.81-4.02) | 4.34(4.31 - 4.38) | 3.48(3.45 - 3.51) | 429.69(428.48-430.89) | 464.94(464.53 - 465.34) | 393.52(393.14 - 393.89) |
| 2003 | 3.93(3.82-4.03) | 4.36(4.33 - 4.4) | 3.47(3.44 - 3.51) | 434.77(433.56-435.97) | 471.3(470.9 - 471.71) | 397.33(396.95 - 397.7) |
| 2004 | 3.92(3.82-4.02) | 4.38(4.34 - 4.41) | 3.45(3.42 - 3.48) | 438.98(437.78-440.18) | 476.95(476.55 - 477.36) | 400.12(399.75 - 400.5) |
| 2005 | 3.9(3.8-4.01) | 4.38(4.35 - 4.42) | 3.41(3.38 - 3.44) | 442.55(441.35-443.74) | 482.15(481.75 - 482.55) | 402.09(401.72 - 402.46) |
| 2006 | 3.86(3.76-3.96) | 4.35(4.32 - 4.39) | 3.36(3.33 - 3.39) | 444.88(443.68-446.07) | 485.38(484.98 - 485.78) | 403.58(403.21 - 403.95) |
| 2007 | 3.82(3.73-3.92) | 4.33(4.3 - 4.37) | 3.31(3.28 - 3.34) | 448.54(447.35-449.72) | 490.88(490.48 - 491.28) | 405.44(405.07 - 405.81) |
| 2008 | 3.8(3.7-3.9) | 4.3(4.27 - 4.34) | 3.28(3.25 - 3.31) | 453.4(452.22-454.58) | 496.32(495.92 - 496.72) | 409.77(409.4 - 410.13) |
| 2009 | 3.78(3.68-3.87) | 4.27(4.24 - 4.31) | 3.27(3.24 - 3.3) | 458.75(457.58-459.93) | 501.72(501.32 - 502.12) | 415.12(414.76 - 415.49) |
| 2010 | 3.74(3.65-3.84) | 4.24(4.21 - 4.28) | 3.24(3.21 - 3.27) | 462.87(461.7-464.05) | 506.96(506.57 - 507.36) | 418.14(417.78 - 418.5) |
| 2011 | 3.7(3.61-3.8) | 4.22(4.18 - 4.25) | 3.19(3.16 - 3.21) | 466.5(465.33-467.67) | 512.1(511.71 - 512.5) | 420.26(419.9 - 420.62) |
| 2012 | 3.7(3.6-3.79) | 4.2(4.17 - 4.24) | 3.18(3.15 - 3.21) | 472.7(471.53-473.86) | 518.92(518.53 - 519.32) | 425.86(425.5 - 426.22) |
| 2013 | 3.72(3.63-3.81) | 4.22(4.18 - 4.25) | 3.22(3.19 - 3.25) | 481.11(479.94-482.28) | 527.49(527.1 - 527.89) | 434.13(433.77 - 434.49) |
| 2014 | 3.74(3.65-3.83) | 4.22(4.19 - 4.25) | 3.25(3.22 - 3.28) | 488.7(487.53-489.88) | 535.3(534.91 - 535.7) | 441.5(441.14 - 441.86) |
| 2015 | 3.77(3.68-3.86) | 4.25(4.22 - 4.28) | 3.28(3.25 - 3.31) | 496.07(494.89-497.25) | 543.21(542.81 - 543.61) | 448.3(447.94 - 448.67) |
| 2016 | 3.84(3.75-3.93) | 4.33(4.3 - 4.37) | 3.34(3.31 - 3.37) | 506.85(505.67-508.03) | 555.11(554.71 - 555.51) | 457.91(457.54 - 458.27) |
| 2017 | 3.89(3.8-3.98) | 4.39(4.36 - 4.42) | 3.38(3.35 - 3.41) | 517.79(516.6-518.98) | 566.84(566.44 - 567.25) | 468.01(467.65 - 468.38) |
| 2018 | 3.93(3.84-4.03) | 4.45(4.41 - 4.48) | 3.42(3.39 - 3.44) | 528.81(527.61-530.01) | 578.87(578.47 - 579.28) | 477.99(477.62 - 478.36) |
| 2019 | 3.97(3.88-4.07) | 4.49(4.46 - 4.53) | 3.45(3.42 - 3.48) | 539.47(538.26-540.67) | 590.25(589.85 - 590.66) | 487.9(487.52 - 488.27) |
| 2020 | 3.99(3.9-4.08) | 4.51(4.48 - 4.54) | 3.46(3.43 - 3.49) | 556.98(555.76-558.2) | 608.95(608.54 - 609.37) | 504.2(503.82 - 504.58) |
| 2021 | 4.03(3.93-4.12) | 4.55(4.52 - 4.59) | 3.49(3.46 - 3.52) | 569.45(568.22-570.68) | 622.64(622.22 - 623.05) | 515.44(515.06 - 515.82) |
| 2022 | 4.05(3.86-4.23) | 4.58(4.45 - 4.72) | 3.5(3.39 - 3.61) | 584.19(566.06-602.33) | 638.03(624.25 - 651.82) | 529.74(518.92 - 540.56) |
| 2023 | 4.07(3.84-4.3) | 4.62(4.43 - 4.81) | 3.52(3.36 - 3.68) | 599.03(575.16-622.9) | 654.06(632.84 - 675.27) | 543.33(526.35 - 560.3) |
| 2024 | 4.1(3.81-4.39) | 4.65(4.38 - 4.92) | 3.54(3.32 - 3.76) | 614.27(582.18-646.37) | 670.56(639.56 - 701.55) | 557.14(532.09 - 582.2) |
| 2025 | 4.12(3.76-4.49) | 4.68(4.33 - 5.04) | 3.55(3.25 - 3.85) | 629.89(587.4-672.39) | 687.49(644.71 - 730.28) | 571.15(536.38 - 605.92) |
| 2026 | 4.14(3.69-4.59) | 4.71(4.25 - 5.16) | 3.56(3.18 - 3.94) | 645.82(590.9-700.74) | 704.76(648.26 - 761.26) | 585.28(539.23 - 631.33) |
| 2027 | 4.16(3.61-4.71) | 4.73(4.16 - 5.29) | 3.57(3.1 - 4.04) | 662.19(592.84-731.55) | 722.47(650.3 - 794.63) | 599.64(540.72 - 658.56) |
| 2028 | 4.18(3.51-4.84) | 4.75(4.07 - 5.43) | 3.58(3.01 - 4.14) | 679.2(593.5-764.9) | 740.79(651.06 - 830.52) | 614.38(541.06 - 687.7) |
| 2029 | 4.19(3.41-4.97) | 4.77(3.96 - 5.58) | 3.58(2.91 - 4.25) | 696.92(592.95-800.89) | 759.82(650.61 - 869.03) | 629.54(540.28 - 718.8) |
| 2030 | 4.22(3.31-5.12) | 4.79(3.85 - 5.73) | 3.59(2.81 - 4.37) | 715.38(591.15-839.6) | 779.59(648.91 - 910.26) | 645.05(538.29 - 751.81) |
| 2031 | 4.24(3.2-5.28) | 4.81(3.73 - 5.9) | 3.6(2.7 - 4.49) | 734.47(587.9-881.05) | 800.01(645.77 - 954.25) | 660.78(534.9 - 786.66) |
| 2032 | 4.27(3.08-5.46) | 4.85(3.6 - 6.09) | 3.6(2.58 - 4.63) | 754.26(583.05-925.47) | 821.08(641.03 - 1001.12) | 676.73(530.02 - 823.45) |
| 2033 | 4.31(2.95-5.66) | 4.88(3.48 - 6.29) | 3.62(2.47 - 4.77) | 774.8(576.62-972.99) | 842.74(634.62 - 1050.86) | 693.03(523.73 - 862.33) |
| 2034 | 4.35(2.82-5.87) | 4.92(3.34 - 6.5) | 3.63(2.34 - 4.92) | 796.16(568.52-1023.8) | 864.96(626.44 - 1103.49) | 709.76(516.07 - 903.45) |
| 2035 | 4.39(2.69-6.1) | 4.96(3.2 - 6.73) | 3.65(2.22 - 5.08) | 818.4(558.66-1078.14) | 887.75(616.38 - 1159.11) | 726.95(506.96 - 946.93) |
| 2036 | 4.45(2.54-6.35) | 5.01(3.05 - 6.97) | 3.67(2.09 - 5.26) | 841.46(546.79-1136.13) | 911.01(604.25 - 1217.77) | 744.47(496.23 - 992.7) |
| 2037 | 4.51(2.39-6.63) | 5.06(2.9 - 7.22) | 3.7(1.95 - 5.45) | 865.34(532.67-1198.02) | 934.72(589.86 - 1279.58) | 762.24(483.68 - 1040.79) |
| 2038 | 4.57(2.22-6.92) | 5.11(2.73 - 7.49) | 3.73(1.81 - 5.65) | 890.14(516.16-1264.11) | 958.86(573.11 - 1344.61) | 780.33(469.31 - 1091.34) |
| 2039 | 4.64(2.05-7.24) | 5.16(2.55 - 7.77) | 3.76(1.66 - 5.86) | 915.98(497.15-1334.8) | 983.48(553.94 - 1413.02) | 798.83(453.1 - 1144.56) |
| 2040 | 4.72(1.85-7.58) | 5.22(2.37 - 8.06) | 3.79(1.5 - 6.07) | 942.97(475.42-1410.53) | 1008.67(532.24 - 1485.11) | 817.76(434.94 - 1200.58) |

**ASMR: age-standardized mortality rate;**

**ASDR: age-standardized DALYs rate;**

**DALYs: disability-adjusted life years.**

**Supplementary Table 4.** Age-standardized DALYs rates of diabetes mellitus due to metabolic factors with frontier analysis across all countries and territories.

| **Location** | **SDI** | **Rate of DALYs** | **Frontier DALYs** | **Effective difference** | **Effective difference rank (Age-standardized DALYs rank)** |
| --- | --- | --- | --- | --- | --- |
| Afghanistan | 0.337199997837472 | 1179.807(836.903 to 1606.04) | 223.023 | 956.784 | 171 (174) |
| Albania | 0.706849790878611 | 221.592(150.212 to 315.693) | 161.899 | 59.694 | 1 (1) |
| Algeria | 0.659500924324217 | 614.834(430.394 to 841.092) | 162.26 | 452.575 | 117 (110) |
| American Samoa | 0.723727533059592 | 2842.49(2149.186 to 3725.364) | 161.813 | 2680.677 | 200 (200) |
| Andorra | 0.869444112735696 | 349.96(240.93 to 488.142) | 139.59 | 210.37 | 38 (32) |
| Angola | 0.453721949492174 | 835.762(626.822 to 1102.281) | 219.141 | 616.621 | 146 (150) |
| Antigua and Barbuda | 0.749886887369379 | 922.799(684.315 to 1220.154) | 140.074 | 782.726 | 161 (158) |
| Argentina | 0.723122972582249 | 348.951(263.56 to 460.603) | 159.891 | 189.06 | 30 (31) |
| Armenia | 0.701833194147139 | 450.092(344.991 to 594.678) | 160.828 | 289.263 | 66 (59) |
| Australia | 0.844252813727575 | 232.731(170.009 to 313.87) | 139.467 | 93.264 | 4 (3) |
| Austria | 0.853837004256539 | 224.26(153.659 to 313.985) | 139.805 | 84.455 | 2 (2) |
| Azerbaijan | 0.694851274415291 | 454.579(338.339 to 608.675) | 162.447 | 292.132 | 67 (60) |
| Bahamas | 0.805020667882124 | 1121.388(857.01 to 1456.118) | 138.981 | 982.407 | 173 (171) |
| Bahrain | 0.753043204117567 | 950.014(709.047 to 1256.224) | 139.665 | 810.349 | 164 (162) |
| Bangladesh | 0.492420884955353 | 743.687(558.122 to 991.276) | 217.839 | 525.848 | 131 (136) |
| Barbados | 0.746748764251474 | 1037.712(791.558 to 1355.181) | 139.657 | 898.055 | 168 (168) |
| Belarus | 0.784484710906639 | 279.728(215.222 to 368.059) | 139.147 | 140.581 | 11 (10) |
| Belgium | 0.853654015777112 | 367.856(244.682 to 530.179) | 138.908 | 228.948 | 44 (35) |
| Belize | 0.610229002222948 | 1100.513(883.079 to 1392.281) | 161.786 | 938.727 | 170 (169) |
| Benin | 0.373486574239249 | 699.03(516.891 to 940.684) | 223.318 | 475.712 | 121 (127) |
| Bermuda | 0.821365422389484 | 523.386(376.912 to 708.637) | 139.309 | 384.077 | 102 (85) |
| Bhutan | 0.473062378179926 | 538.765(394.888 to 733.584) | 219.336 | 319.43 | 74 (91) |
| Bolivia (Plurinational State of) | 0.5990107988623 | 529.47(398.673 to 708.47) | 163.726 | 365.745 | 95 (88) |
| Bosnia and Herzegovina | 0.72307789321024 | 406.552(293.545 to 562.37) | 162.825 | 243.727 | 48 (45) |
| Botswana | 0.642721628921122 | 469.641(350.598 to 622.014) | 161.815 | 307.826 | 72 (64) |
| Brazil | 0.65304388724724 | 541.048(435.182 to 668.864) | 162.439 | 378.609 | 100 (93) |
| Brunei Darussalam | 0.810234366947283 | 1237.46(931.272 to 1608.093) | 138.919 | 1098.541 | 177 (176) |
| Bulgaria | 0.768150939342518 | 437.065(337.795 to 568.772) | 140.754 | 296.31 | 68 (56) |
| Burkina Faso | 0.285118401552141 | 593.553(446.043 to 778.334) | 232.936 | 360.616 | 92 (107) |
| Burundi | 0.289374364823265 | 559.905(404.036 to 783.612) | 229.429 | 330.477 | 80 (98) |
| Cabo Verde | 0.53353453903661 | 615.87(450.615 to 831.707) | 217.044 | 398.826 | 103 (111) |
| Cambodia | 0.473621490708013 | 518.699(374.74 to 687.911) | 216.948 | 301.751 | 70 (81) |
| Cameroon | 0.479691223419746 | 735.609(537.038 to 986.336) | 218.304 | 517.305 | 128 (134) |
| Canada | 0.873170679735405 | 370.927(271.727 to 495.351) | 139.439 | 231.488 | 45 (37) |
| Central African Republic | 0.309167689992842 | 1148.923(833.774 to 1528.552) | 225.834 | 923.089 | 169 (172) |
| Chad | 0.240436018662427 | 578.885(433.676 to 764.734) | 237.241 | 341.644 | 87 (103) |
| Chile | 0.771514715903929 | 302.461(215.027 to 414.266) | 140.35 | 162.111 | 19 (16) |
| China | 0.721629759719777 | 490.525(336.573 to 677.564) | 162.488 | 328.037 | 78 (71) |
| Colombia | 0.655442912568827 | 538.693(387.561 to 736.424) | 163.105 | 375.588 | 98 (90) |
| Comoros | 0.475978688247514 | 664.629(492.911 to 883.308) | 217.857 | 446.772 | 116 (119) |
| Congo | 0.583075236262154 | 943.263(689.671 to 1265.185) | 161.142 | 782.121 | 160 (160) |
| Cook Islands | 0.779109954659683 | 2482.805(1863.898 to 3261.775) | 138.867 | 2343.938 | 199 (198) |
| Costa Rica | 0.700340476999325 | 666.791(480.327 to 913.669) | 163.031 | 503.761 | 125 (121) |
| Coted'Ivoire | 0.425941883067361 | 665.195(493.336 to 879.48) | 220.342 | 444.853 | 115 (120) |
| Croatia | 0.798341026936276 | 298.381(213.064 to 414.727) | 139.322 | 159.059 | 17 (15) |
| Cuba | 0.668729864363244 | 523.723(363.248 to 730.277) | 159.936 | 363.787 | 93 (86) |
| Cyprus | 0.835630545002355 | 360.253(257.829 to 490.718) | 140.435 | 219.818 | 41 (34) |
| Czechia | 0.828450433122785 | 323.574(234.253 to 446.919) | 138.915 | 184.659 | 27 (23) |
| Democratic People's Republic of Korea | 0.569854634067777 | 507.981(365.69 to 690.952) | 161.172 | 346.809 | 89 (78) |
| Democratic Republic of the Congo | 0.38317984920136 | 727.574(547.566 to 961.241) | 219.371 | 508.204 | 126 (133) |
| Denmark | 0.896424204101212 | 267.152(185.225 to 365.17) | 139.413 | 127.739 | 9 (8) |
| Djibouti | 0.4879583707169 | 546.986(391.516 to 755.118) | 218.616 | 328.37 | 79 (94) |
| Dominica | 0.746967184749286 | 1245.394(943.175 to 1642.423) | 155.205 | 1090.189 | 176 (177) |
| Dominican Republic | 0.619388201281128 | 1011.462(745.951 to 1334.972) | 159.857 | 851.605 | 166 (166) |
| Ecuador | 0.66101705262103 | 584.963(442.873 to 751.134) | 163.088 | 421.875 | 109 (105) |
| Egypt | 0.6067870937016 | 685.144(519.577 to 893.681) | 162.604 | 522.54 | 129 (122) |
| El Salvador | 0.563775187564695 | 967.955(749.582 to 1226.71) | 162.759 | 805.195 | 163 (163) |
| Equatorial Guinea | 0.657857455522296 | 935.512(660.295 to 1280.17) | 163.129 | 772.383 | 159 (159) |
| Eritrea | 0.403863942828898 | 814.346(582.58 to 1139.102) | 217.313 | 597.033 | 142 (147) |
| Estonia | 0.844917787360624 | 424.108(316.963 to 567.587) | 139.493 | 284.615 | 63 (52) |
| Eswatini | 0.58545971329224 | 975.818(644.542 to 1390.648) | 161.811 | 814.007 | 165 (165) |
| Ethiopia | 0.358823295416803 | 524.742(421.739 to 635.998) | 223.457 | 301.285 | 69 (87) |
| Fiji | 0.675051630810876 | 3309.288(2570.288 to 4301.04) | 163.817 | 3145.47 | 203 (203) |
| Finland | 0.859831367662218 | 513.436(347.088 to 726.925) | 139.262 | 374.174 | 97 (80) |
| France | 0.838364875196954 | 234.728(160.918 to 326.176) | 139.721 | 95.007 | 5 (4) |
| Gabon | 0.634691392755259 | 1030.648(745.425 to 1406.972) | 159.887 | 870.761 | 167 (167) |
| Gambia | 0.40971415951073 | 647.181(482.017 to 854.099) | 217.35 | 429.832 | 111 (117) |
| Georgia | 0.732473604476306 | 562.489(423.655 to 751.648) | 160.777 | 401.713 | 104 (99) |
| Germany | 0.902957091057572 | 315.82(219.814 to 428.247) | 139.312 | 176.508 | 26 (21) |
| Ghana | 0.564930389658009 | 712.23(537.327 to 947.413) | 162.006 | 550.225 | 136 (129) |
| Greece | 0.791854407912829 | 408.526(269.118 to 576.156) | 139.856 | 268.669 | 56 (46) |
| Greenland | 0.82621033634361 | 260.358(192.503 to 339.764) | 139.961 | 120.397 | 8 (7) |
| Grenada | 0.668993028326993 | 1354.42(1073.535 to 1729.058) | 159.867 | 1194.552 | 181 (181) |
| Guam | 0.803982202863145 | 943.321(705.319 to 1238.353) | 139.691 | 803.631 | 162 (161) |
| Guatemala | 0.539972423611408 | 1536.171(1268.245 to 1884.465) | 219.488 | 1316.683 | 184 (184) |
| Guinea | 0.33640129335069 | 585.239(442.974 to 759.266) | 220.414 | 364.825 | 94 (106) |
| Guinea-Bissau | 0.353109621364536 | 883.062(645.004 to 1178.618) | 220.038 | 663.023 | 151 (155) |
| Guyana | 0.650812335285137 | 2065.152(1571.75 to 2698.272) | 161.845 | 1903.308 | 192 (192) |
| Haiti | 0.448278284992742 | 1467.783(1087.967 to 1934.697) | 220.073 | 1247.71 | 183 (183) |
| Honduras | 0.513037248307344 | 782.878(553.717 to 1092.328) | 221.032 | 561.847 | 139 (142) |
| Hungary | 0.790754768173338 | 335.037(240.458 to 454.548) | 139.767 | 195.27 | 32 (27) |
| Iceland | 0.876361679802766 | 328.136(218.012 to 463.85) | 139.555 | 188.58 | 29 (24) |
| India | 0.575401649396992 | 620.227(480.018 to 794.434) | 163.132 | 457.095 | 118 (113) |
| Indonesia | 0.656868336261681 | 498.214(395.63 to 609.919) | 163.803 | 334.41 | 83 (74) |
| Iran (Islamic Republic of) | 0.697207397661899 | 431.163(317.751 to 575.017) | 163.127 | 268.036 | 55 (53) |
| Iraq | 0.662626230762701 | 1121.068(804.353 to 1510.69) | 159.859 | 961.209 | 172 (170) |
| Ireland | 0.873753849787408 | 282.54(188.977 to 397.259) | 139.228 | 143.312 | 12 (11) |
| Israel | 0.809011651557631 | 315.097(224.232 to 429.259) | 140.318 | 174.779 | 24 (20) |
| Italy | 0.805773533613512 | 284.364(197.201 to 399.162) | 138.523 | 145.841 | 13 (12) |
| Jamaica | 0.683263063531091 | 888.913(681.241 to 1157.467) | 163.031 | 725.881 | 156 (156) |
| Japan | 0.871241812572128 | 356.462(243.464 to 499.325) | 140.554 | 215.908 | 39 (33) |
| Jordan | 0.725307226832443 | 780.125(556.281 to 1047.112) | 161.104 | 619.021 | 147 (140) |
| Kazakhstan | 0.725144495100349 | 505.073(355.046 to 697.192) | 162.868 | 342.204 | 88 (76) |
| Kenya | 0.523768077100117 | 405.501(334.017 to 494.709) | 218.175 | 187.326 | 28 (44) |
| Kiribati | 0.527186582842681 | 3281.128(2417.967 to 4383.896) | 217.996 | 3063.131 | 202 (202) |
| Kuwait | 0.846651054742869 | 862.989(568.537 to 1205.522) | 139.72 | 723.268 | 155 (152) |
| Kyrgyzstan | 0.603979328412055 | 384.929(281.207 to 519.429) | 162.586 | 222.343 | 42 (39) |
| Lao People's Democratic Republic | 0.489136091456599 | 625.743(464.53 to 819.115) | 219.255 | 406.488 | 106 (115) |
| Latvia | 0.830663516299797 | 477(380.602 to 611.553) | 139.242 | 337.758 | 85 (67) |
| Lebanon | 0.74474635096338 | 791.506(562.307 to 1092.715) | 151.151 | 640.355 | 149 (143) |
| Lesotho | 0.510393065681381 | 735.901(520.614 to 1007.324) | 219.255 | 516.645 | 127 (135) |
| Liberia | 0.352442451643965 | 721.906(521.091 to 978.545) | 219.721 | 502.185 | 124 (130) |
| Libya | 0.725771398888229 | 722.451(514.553 to 980.039) | 161.83 | 560.621 | 138 (131) |
| Lithuania | 0.856484049109405 | 396.63(310.258 to 514.887) | 140.037 | 256.593 | 52 (42) |
| Luxembourg | 0.884428955125693 | 312.982(205.931 to 448.407) | 139.503 | 173.479 | 23 (18) |
| Madagascar | 0.40024694263784 | 511.306(381.373 to 667.749) | 224.033 | 287.273 | 65 (79) |
| Malawi | 0.384553633730301 | 559.261(421.66 to 737.564) | 223.274 | 335.986 | 84 (96) |
| Malaysia | 0.742523828414751 | 518.839(394.211 to 678.66) | 158.271 | 360.567 | 91 (83) |
| Maldives | 0.650886627343107 | 368.741(273.071 to 493.504) | 163.284 | 205.458 | 36 (36) |
| Mali | 0.268579940925684 | 841.552(625.643 to 1123.151) | 238.135 | 603.416 | 144 (151) |
| Malta | 0.801585033998971 | 462.88(319.528 to 636.595) | 139.376 | 323.504 | 76 (62) |
| Marshall Islands | 0.574091128177309 | 3857.044(2772.657 to 5207.119) | 162.109 | 3694.935 | 204 (204) |
| Mauritania | 0.498945100442996 | 421.632(311.909 to 559.548) | 217.992 | 203.64 | 34 (51) |
| Mauritius | 0.718260445552653 | 1753.682(1524.263 to 2063.104) | 164.878 | 1588.804 | 186 (186) |
| Mexico | 0.664575304259183 | 1371.246(1130.93 to 1657.202) | 159.858 | 1211.388 | 182 (182) |
| Micronesia (Federated States of) | 0.587534967103163 | 2369.623(1760.765 to 3111.03) | 161.732 | 2207.891 | 195 (195) |
| Monaco | 0.908262830989574 | 312.996(208.813 to 441.86) | 140.271 | 172.725 | 21 (19) |
| Mongolia | 0.617621564638127 | 433.185(323.992 to 574.206) | 163.108 | 270.077 | 58 (54) |
| Montenegro | 0.795800584021403 | 400.206(291.173 to 541.404) | 139.284 | 260.922 | 53 (43) |
| Morocco | 0.562698300927195 | 868.396(591.101 to 1212.043) | 160.111 | 708.285 | 154 (154) |
| Mozambique | 0.326462613636377 | 794.923(568.753 to 1090.871) | 218.611 | 576.311 | 140 (145) |
| Myanmar | 0.533900839611906 | 973.218(746.101 to 1255.556) | 216.526 | 756.692 | 158 (164) |
| Namibia | 0.617564872149664 | 501.963(359.052 to 686.603) | 162.711 | 339.252 | 86 (75) |
| Nauru | 0.625177833565553 | 3024.596(2244.955 to 3979.961) | 162.454 | 2862.142 | 201 (201) |
| Nepal | 0.43317463481343 | 744.975(545.765 to 998.9) | 220.495 | 524.48 | 130 (137) |
| Netherlands | 0.888464256397732 | 292.107(196.946 to 408.012) | 139.223 | 152.884 | 16 (14) |
| New Zealand | 0.849442498970719 | 291.263(219.223 to 382.607) | 138.523 | 152.739 | 15 (13) |
| Nicaragua | 0.523958472235925 | 799.028(597.837 to 1041.975) | 219.149 | 579.879 | 141 (146) |
| Niger | 0.168072773803995 | 479.466(350.725 to 647.771) | 333.106 | 146.36 | 14 (68) |
| Nigeria | 0.503390832763244 | 454.806(352.669 to 586.379) | 218.317 | 236.489 | 46 (61) |
| Niue | 0.726222049709539 | 2469.393(1822.944 to 3266.069) | 163.51 | 2305.883 | 197 (197) |
| North Macedonia | 0.750629703270047 | 412.644(304.232 to 555.057) | 139.932 | 272.712 | 59 (47) |
| Northern Mariana Islands | 0.771535213383281 | 1228.13(939.405 to 1608.614) | 139.355 | 1088.776 | 175 (175) |
| Norway | 0.916132810119013 | 345.071(244.694 to 472.399) | 140.423 | 204.648 | 35 (30) |
| Oman | 0.773391601871737 | 617.954(459.524 to 816.923) | 140.335 | 477.619 | 122 (112) |
| Pakistan | 0.504028688613448 | 865.002(657.334 to 1108.754) | 220.74 | 644.262 | 150 (153) |
| Palau | 0.75404693141209 | 2434.38(1836.106 to 3187.327) | 139.243 | 2295.138 | 196 (196) |
| Palestine | 0.631011665054344 | 599.357(443.352 to 782.082) | 162.893 | 436.464 | 113 (109) |
| Panama | 0.708864827518009 | 696.446(520.071 to 923.341) | 162.482 | 533.964 | 134 (125) |
| Papua New Guinea | 0.417797443084353 | 1896.318(1456.764 to 2445.453) | 221.091 | 1675.227 | 189 (190) |
| Paraguay | 0.635718098852038 | 695.635(537.75 to 900.489) | 163.089 | 532.546 | 132 (124) |
| Peru | 0.662054036755862 | 322.516(243.275 to 424.587) | 161.882 | 160.635 | 18 (22) |
| Philippines | 0.651219328702707 | 637.974(542.534 to 750.72) | 162.797 | 475.178 | 120 (116) |
| Poland | 0.812042808667712 | 377.422(286.518 to 489.944) | 139.604 | 237.818 | 47 (38) |
| Portugal | 0.744151850665712 | 466.328(314.576 to 652.41) | 142.674 | 323.655 | 77 (63) |
| Puerto Rico | 0.825525846914029 | 1177.015(903.373 to 1525.292) | 139.554 | 1037.461 | 174 (173) |
| Qatar | 0.846860584038945 | 746.474(526.194 to 1020.978) | 138.823 | 607.651 | 145 (138) |
| Republic of Korea | 0.886675266756997 | 685.217(465.182 to 965.956) | 139.266 | 545.951 | 135 (123) |
| Republic of Moldova | 0.732214875427507 | 480.205(361.721 to 629.555) | 160.116 | 320.09 | 75 (69) |
| Romania | 0.768453864365965 | 243.454(172.241 to 337.363) | 140.501 | 102.952 | 6 (5) |
| Russian Federation | 0.808536004906566 | 344.294(268.233 to 440.458) | 138.797 | 205.497 | 37 (29) |
| Rwanda | 0.435588705789772 | 476.567(311.423 to 706.66) | 220.273 | 256.294 | 51 (66) |
| Saint Kitts and Nevis | 0.754987054945241 | 896.917(670.105 to 1208.538) | 140.666 | 756.252 | 157 (157) |
| Saint Lucia | 0.672509735365292 | 1335.073(1031.347 to 1738.46) | 159.866 | 1175.207 | 180 (180) |
| Saint Vincent and the Grenadines | 0.637195962512539 | 1574.238(1255.186 to 1982.156) | 161.838 | 1412.401 | 185 (185) |
| Samoa | 0.593392769469481 | 2125.218(1570.401 to 2791.244) | 161.834 | 1963.384 | 193 (193) |
| San Marino | 0.888005473958106 | 311.647(204.153 to 445.138) | 138.692 | 172.955 | 22 (17) |
| Sao Tome and Principe | 0.505413746708186 | 497.629(351.973 to 684) | 216.144 | 281.485 | 61 (73) |
| Saudi Arabia | 0.815143493214829 | 835.278(610.159 to 1104.279) | 140.03 | 695.248 | 153 (149) |
| Senegal | 0.408054192634391 | 702.164(524.108 to 917.498) | 221.083 | 481.081 | 123 (128) |
| Serbia | 0.792416293685483 | 392.176(284.707 to 534.59) | 139.174 | 253.002 | 50 (40) |
| Seychelles | 0.730150774629382 | 763.153(562.641 to 1013.366) | 161.849 | 601.304 | 143 (139) |
| Sierra Leone | 0.3586658807038 | 571.85(427.825 to 771.535) | 219.6 | 352.25 | 90 (102) |
| Singapore | 0.856097766112729 | 520.679(339.246 to 762.256) | 139.38 | 381.299 | 101 (84) |
| Slovakia | 0.810610529921693 | 270.349(194.023 to 371.671) | 139.855 | 130.494 | 10 (9) |
| Slovenia | 0.842430731281465 | 255.007(181.17 to 354.275) | 139.285 | 115.722 | 7 (6) |
| Solomon Islands | 0.429360316406646 | 2529.782(1868.881 to 3346.786) | 219.7 | 2310.082 | 198 (199) |
| Somalia | 0.0776881089723748 | 725.139(515.233 to 988.099) | 635.945 | 89.194 | 3 (132) |
| South Africa | 0.679626598156588 | 698.14(580.519 to 825.218) | 165.145 | 532.995 | 133 (126) |
| South Sudan | 0.278371125484101 | 659.023(477.415 to 901.642) | 236.616 | 422.407 | 110 (118) |
| Spain | 0.769283697979998 | 416.357(275.003 to 588.152) | 138.791 | 277.566 | 60 (49) |
| Sri Lanka | 0.70153493519423 | 830.14(602.82 to 1121.221) | 162.71 | 667.43 | 152 (148) |
| Sudan | 0.54194973514933 | 518.816(373.394 to 707.391) | 216.96 | 301.856 | 71 (82) |
| Suriname | 0.63366573910901 | 1300.506(969.008 to 1742.055) | 162.784 | 1137.722 | 179 (179) |
| Sweden | 0.886880298870342 | 339.691(240.308 to 463.943) | 139.702 | 199.989 | 33 (28) |
| Switzerland | 0.933059110986638 | 420.939(276.075 to 601.209) | 138.807 | 282.132 | 62 (50) |
| Syrian Arab Republic | 0.62300407463119 | 540.99(380.14 to 738.784) | 162.677 | 378.314 | 99 (92) |
| Taiwan (Province of China) | 0.874747053187929 | 507.284(378.883 to 660.104) | 139.31 | 367.974 | 96 (77) |
| Tajikistan | 0.541511187051998 | 437.047(329.828 to 579.276) | 218.172 | 218.875 | 40 (55) |
| Thailand | 0.682547932974593 | 565.295(427.035 to 730.59) | 162.817 | 402.479 | 105 (100) |
| Timor-Leste | 0.444667618881833 | 445.532(325.416 to 592.734) | 220.13 | 225.402 | 43 (57) |
| Togo | 0.408533695477986 | 493.043(358.554 to 655.97) | 227.272 | 265.772 | 54 (72) |
| Tokelau | 0.68642562083005 | 2176.846(1610.746 to 2862.223) | 161.904 | 2014.943 | 194 (194) |
| Tonga | 0.626349935807987 | 1963.623(1479.18 to 2598.247) | 162.532 | 1801.092 | 191 (191) |
| Trinidad and Tobago | 0.768763253980677 | 1782.416(1405.25 to 2305.978) | 140.186 | 1642.23 | 188 (187) |
| Tunisia | 0.682432215876962 | 581.151(404.309 to 820.711) | 160.227 | 420.925 | 108 (104) |
| Turkey | 0.712692673098478 | 448.846(318.689 to 612.868) | 163.11 | 285.737 | 64 (58) |
| Turkmenistan | 0.682160775869611 | 620.688(492.29 to 783.822) | 162.484 | 458.204 | 119 (114) |
| Tuvalu | 0.576620529344684 | 1861.17(1405.371 to 2410.245) | 162.376 | 1698.794 | 190 (189) |
| Uganda | 0.423261181495743 | 552.735(392.008 to 783.721) | 220.076 | 332.659 | 82 (95) |
| Ukraine | 0.760773912846782 | 334.101(241.883 to 444.447) | 138.964 | 195.137 | 31 (26) |
| United Arab Emirates | 0.849317734035032 | 473.385(328.561 to 652.197) | 140.776 | 332.609 | 81 (65) |
| United Kingdom | 0.859000181772086 | 559.296(377.594 to 780.002) | 139.23 | 420.066 | 107 (97) |
| United Republic of Tanzania | 0.446568273334685 | 487.457(359.087 to 647.906) | 218.299 | 269.159 | 57 (70) |
| United States Virgin Islands | 0.821830853383701 | 1262.906(919.225 to 1704.799) | 140.076 | 1122.83 | 178 (178) |
| United States of America | 0.862448354015145 | 571.365(445.681 to 729.643) | 139.646 | 431.719 | 112 (101) |
| Uruguay | 0.719283444839432 | 330.927(245.177 to 444.502) | 163.163 | 167.764 | 20 (25) |
| Uzbekistan | 0.662621694181816 | 598.356(463.563 to 780.769) | 159.937 | 438.419 | 114 (108) |
| Vanuatu | 0.473100705820327 | 1843.482(1389.534 to 2399.562) | 215.957 | 1627.525 | 187 (188) |
| Venezuela (Bolivarian Republic of) | 0.596513058857335 | 794.237(611.423 to 1032.38) | 163.16 | 631.078 | 148 (144) |
| Viet Nam | 0.627933720680671 | 415.344(315.974 to 548.717) | 162.8 | 252.543 | 49 (48) |
| Yemen | 0.450376375244811 | 395.782(274.442 to 546.364) | 219.983 | 175.798 | 25 (41) |
| Zambia | 0.505948954434909 | 780.296(575.948 to 1039.923) | 221.463 | 558.833 | 137 (141) |
| Zimbabwe | 0.473819486202707 | 535.032(387.495 to 705.15) | 220.264 | 314.768 | 73 (89) |

**DALYs: disability-adjusted life years;**

**SDI: Socio-Demographic Index.**

**Supplementary Table 5.** Age-standardized mortality rates of diabetes mellitus due to metabolic factors with frontier analysis across all countries and territories

| **Location** | **SDI** | **Rate of mortality** | **Frontier mortality** | **Effective difference** | **Effective difference rank (Age-standardized mortality rank)** |
| --- | --- | --- | --- | --- | --- |
| Afghanistan | 0.337199997837472 | 7.222(4.493 to 11.45) | 1.901 | 5.321 | 131 (140) |
| Albania | 0.706849790878611 | 0.681(0.475 to 0.963) | 0.68 | 0.001 | 3 (14) |
| Algeria | 0.659500924324217 | 2.284(1.615 to 3.143) | 0.746 | 1.538 | 54 (54) |
| American Samoa | 0.723727533059592 | 26.116(18.857 to 35.231) | 0.707 | 25.409 | 198 (198) |
| Andorra | 0.869444112735696 | 1.03(0.609 to 1.521) | 0.406 | 0.624 | 26 (22) |
| Angola | 0.453721949492174 | 8.217(5.492 to 11.725) | 1.899 | 6.318 | 147 (151) |
| Antigua and Barbuda | 0.749886887369379 | 6.013(5.214 to 6.929) | 0.636 | 5.378 | 132 (119) |
| Argentina | 0.723122972582249 | 2.418(2.123 to 2.76) | 0.724 | 1.694 | 58 (59) |
| Armenia | 0.701833194147139 | 3.337(2.802 to 3.972) | 0.739 | 2.598 | 79 (75) |
| Australia | 0.844252813727575 | 1.245(1.095 to 1.406) | 0.492 | 0.753 | 31 (27) |
| Austria | 0.853837004256539 | 0.645(0.571 to 0.733) | 0.443 | 0.201 | 16 (13) |
| Azerbaijan | 0.694851274415291 | 3.191(2.201 to 4.189) | 0.735 | 2.456 | 77 (74) |
| Bahamas | 0.805020667882124 | 9.243(7.058 to 12.08) | 0.461 | 8.782 | 164 (160) |
| Bahrain | 0.753043204117567 | 7.025(5.301 to 8.981) | 0.611 | 6.413 | 149 (138) |
| Bangladesh | 0.492420884955353 | 4.887(3.434 to 6.855) | 1.542 | 3.345 | 94 (104) |
| Barbados | 0.746748764251474 | 8.671(6.516 to 11.446) | 0.671 | 8 | 159 (153) |
| Belarus | 0.784484710906639 | 2.097(1.66 to 2.595) | 0.46 | 1.637 | 57 (52) |
| Belgium | 0.853654015777112 | 0.633(0.561 to 0.714) | 0.403 | 0.231 | 18 (11) |
| Belize | 0.610229002222948 | 10.888(9.208 to 12.8) | 0.742 | 10.145 | 174 (172) |
| Benin | 0.373486574239249 | 5.566(3.757 to 7.97) | 1.9 | 3.666 | 100 (110) |
| Bermuda | 0.821365422389484 | 2.395(1.883 to 2.986) | 0.474 | 1.92 | 67 (58) |
| Bhutan | 0.473062378179926 | 3.869(2.417 to 5.896) | 1.636 | 2.233 | 73 (87) |
| Bolivia (Plurinational State of) | 0.5990107988623 | 5.267(3.592 to 7.529) | 0.746 | 4.521 | 117 (108) |
| Bosnia and Herzegovina | 0.72307789321024 | 2.573(1.758 to 3.437) | 0.719 | 1.854 | 64 (63) |
| Botswana | 0.642721628921122 | 4.81(3.199 to 6.911) | 0.743 | 4.067 | 110 (102) |
| Brazil | 0.65304388724724 | 4.814(4.582 to 5.044) | 0.737 | 4.078 | 111 (103) |
| Brunei Darussalam | 0.810234366947283 | 10.348(7.807 to 13.326) | 0.529 | 9.819 | 169 (164) |
| Bulgaria | 0.768150939342518 | 3.491(2.891 to 4.163) | 0.473 | 3.018 | 87 (79) |
| Burkina Faso | 0.285118401552141 | 6.736(4.455 to 9.549) | 1.955 | 4.781 | 123 (132) |
| Burundi | 0.289374364823265 | 7.549(4.782 to 11.83) | 1.905 | 5.645 | 138 (146) |
| Cabo Verde | 0.53353453903661 | 4.149(2.811 to 5.99) | 1.434 | 2.715 | 82 (92) |
| Cambodia | 0.473621490708013 | 5.951(3.875 to 9.007) | 1.656 | 4.296 | 114 (117) |
| Cameroon | 0.479691223419746 | 8.741(5.533 to 13.034) | 1.464 | 7.277 | 154 (155) |
| Canada | 0.873170679735405 | 2.097(1.9 to 2.313) | 0.379 | 1.717 | 60 (51) |
| Central African Republic | 0.309167689992842 | 12.709(7.852 to 18.89) | 1.899 | 10.81 | 177 (180) |
| Chad | 0.240436018662427 | 5.745(3.863 to 8.537) | 2.346 | 3.4 | 98 (112) |
| Chile | 0.771514715903929 | 1.248(1.106 to 1.402) | 0.457 | 0.791 | 32 (28) |
| China | 0.721629759719777 | 1.349(1.087 to 1.646) | 0.705 | 0.644 | 27 (30) |
| Colombia | 0.655442912568827 | 2.453(1.979 to 2.983) | 0.754 | 1.699 | 59 (60) |
| Comoros | 0.475978688247514 | 7.515(4.683 to 10.716) | 1.605 | 5.909 | 142 (145) |
| Congo | 0.583075236262154 | 11.738(7.7 to 17.695) | 0.743 | 10.996 | 178 (175) |
| Cook Islands | 0.779109954659683 | 22.525(15.937 to 31.586) | 0.457 | 22.068 | 194 (193) |
| Costa Rica | 0.700340476999325 | 2.909(2.475 to 3.355) | 0.733 | 2.176 | 72 (69) |
| Coted'Ivoire | 0.425941883067361 | 6.768(4.451 to 9.996) | 1.901 | 4.866 | 124 (133) |
| Croatia | 0.798341026936276 | 1.395(1.169 to 1.614) | 0.46 | 0.935 | 37 (33) |
| Cuba | 0.668729864363244 | 1.569(1.305 to 1.872) | 0.738 | 0.831 | 33 (36) |
| Cyprus | 0.835630545002355 | 1.701(1.252 to 2.254) | 0.469 | 1.232 | 45 (39) |
| Czechia | 0.828450433122785 | 1.649(1.368 to 1.946) | 0.466 | 1.183 | 43 (37) |
| Democratic People's Republic of Korea | 0.569854634067777 | 3.374(2.174 to 5.265) | 0.757 | 2.617 | 80 (76) |
| Democratic Republic of the Congo | 0.38317984920136 | 8.233(5.448 to 11.771) | 1.901 | 6.332 | 148 (152) |
| Denmark | 0.896424204101212 | 0.902(0.787 to 1.031) | 0.369 | 0.533 | 22 (18) |
| Djibouti | 0.4879583707169 | 7.336(4.606 to 11.445) | 1.47 | 5.866 | 141 (144) |
| Dominica | 0.746967184749286 | 10.573(7.638 to 14.327) | 0.667 | 9.906 | 170 (168) |
| Dominican Republic | 0.619388201281128 | 7.718(5.522 to 10.48) | 0.748 | 6.971 | 151 (148) |
| Ecuador | 0.66101705262103 | 4.703(3.536 to 6.083) | 0.734 | 3.969 | 107 (99) |
| Egypt | 0.6067870937016 | 5.778(4.185 to 7.768) | 0.778 | 5.001 | 126 (113) |
| El Salvador | 0.563775187564695 | 10.416(7.745 to 13.77) | 0.773 | 9.643 | 168 (165) |
| Equatorial Guinea | 0.657857455522296 | 10.856(6.246 to 17.013) | 0.751 | 10.106 | 173 (171) |
| Eritrea | 0.403863942828898 | 10.687(6.519 to 16.733) | 1.901 | 8.786 | 165 (169) |
| Estonia | 0.844917787360624 | 2.823(2.356 to 3.297) | 0.459 | 2.363 | 75 (67) |
| Eswatini | 0.58545971329224 | 14.998(8.345 to 23.484) | 0.778 | 14.22 | 183 (183) |
| Ethiopia | 0.358823295416803 | 6.12(4.924 to 7.542) | 1.899 | 4.221 | 113 (121) |
| Fiji | 0.675051630810876 | 49.833(36.011 to 67.527) | 0.737 | 49.096 | 204 (204) |
| Finland | 0.859831367662218 | 0.859(0.75 to 0.978) | 0.382 | 0.477 | 21 (17) |
| France | 0.838364875196954 | 0.726(0.644 to 0.815) | 0.501 | 0.226 | 17 (15) |
| Gabon | 0.634691392755259 | 12.358(7.737 to 19.495) | 0.747 | 11.612 | 179 (178) |
| Gambia | 0.40971415951073 | 6.946(4.547 to 10.097) | 1.906 | 5.04 | 128 (136) |
| Georgia | 0.732473604476306 | 3.708(3.173 to 4.321) | 0.715 | 2.993 | 86 (83) |
| Germany | 0.902957091057572 | 1.066(0.939 to 1.201) | 0.364 | 0.703 | 29 (23) |
| Ghana | 0.564930389658009 | 7.946(5.322 to 11.111) | 0.754 | 7.192 | 153 (149) |
| Greece | 0.791854407912829 | 0.637(0.564 to 0.719) | 0.495 | 0.142 | 13 (12) |
| Greenland | 0.82621033634361 | 2.055(1.392 to 2.839) | 0.471 | 1.584 | 56 (49) |
| Grenada | 0.668993028326993 | 12.653(10.184 to 15.354) | 0.734 | 11.92 | 180 (179) |
| Guam | 0.803982202863145 | 6.635(5.295 to 8.334) | 0.484 | 6.151 | 145 (127) |
| Guatemala | 0.539972423611408 | 18.718(15.615 to 22.275) | 1.456 | 17.261 | 185 (186) |
| Guinea | 0.33640129335069 | 6.475(4.419 to 9.257) | 1.9 | 4.575 | 119 (125) |
| Guinea-Bissau | 0.353109621364536 | 10.544(6.875 to 15.453) | 1.904 | 8.641 | 162 (166) |
| Guyana | 0.650812335285137 | 18.784(13.399 to 25.431) | 0.751 | 18.033 | 187 (187) |
| Haiti | 0.448278284992742 | 14.457(9.202 to 21.814) | 1.899 | 12.558 | 181 (181) |
| Honduras | 0.513037248307344 | 4.426(2.741 to 6.807) | 1.459 | 2.967 | 85 (94) |
| Hungary | 0.790754768173338 | 1.772(1.492 to 2.05) | 0.467 | 1.305 | 48 (41) |
| Iceland | 0.876361679802766 | 0.5(0.423 to 0.585) | 0.377 | 0.123 | 10 (6) |
| India | 0.575401649396992 | 4.484(3.848 to 5.11) | 0.757 | 3.727 | 103 (95) |
| Indonesia | 0.656868336261681 | 5.275(4.147 to 6.886) | 0.742 | 4.533 | 118 (109) |
| Iran (Islamic Republic of) | 0.697207397661899 | 1.948(1.71 to 2.178) | 0.737 | 1.211 | 44 (44) |
| Iraq | 0.662626230762701 | 6.021(4.08 to 8.875) | 0.737 | 5.283 | 130 (120) |
| Ireland | 0.873753849787408 | 0.503(0.435 to 0.58) | 0.388 | 0.115 | 9 (8) |
| Israel | 0.809011651557631 | 1.34(1.181 to 1.506) | 0.506 | 0.835 | 34 (29) |
| Italy | 0.805773533613512 | 0.627(0.595 to 0.658) | 0.459 | 0.169 | 14 (10) |
| Jamaica | 0.683263063531091 | 8.703(6.264 to 11.954) | 0.733 | 7.97 | 158 (154) |
| Japan | 0.871241812572128 | 0.501(0.483 to 0.519) | 0.378 | 0.123 | 11 (7) |
| Jordan | 0.725307226832443 | 3.825(2.779 to 5.11) | 0.72 | 3.105 | 88 (86) |
| Kazakhstan | 0.725144495100349 | 1.755(1.455 to 2.129) | 0.715 | 1.039 | 40 (40) |
| Kenya | 0.523768077100117 | 5.78(4.562 to 7.265) | 1.449 | 4.331 | 115 (114) |
| Kiribati | 0.527186582842681 | 47.502(31.886 to 70.057) | 1.482 | 46.02 | 203 (203) |
| Kuwait | 0.846651054742869 | 1.874(1.486 to 2.327) | 0.457 | 1.417 | 51 (42) |
| Kyrgyzstan | 0.603979328412055 | 2.086(1.649 to 2.611) | 0.757 | 1.329 | 50 (50) |
| Lao People's Democratic Republic | 0.489136091456599 | 7.015(4.744 to 9.966) | 1.433 | 5.582 | 137 (137) |
| Latvia | 0.830663516299797 | 4.119(3.47 to 4.745) | 0.46 | 3.659 | 99 (91) |
| Lebanon | 0.74474635096338 | 3.41(2.332 to 4.674) | 0.687 | 2.723 | 83 (77) |
| Lesotho | 0.510393065681381 | 10.927(6.749 to 15.811) | 1.441 | 9.485 | 167 (173) |
| Liberia | 0.352442451643965 | 7.323(4.615 to 11.208) | 1.9 | 5.423 | 133 (142) |
| Libya | 0.725771398888229 | 3.979(2.544 to 5.754) | 0.714 | 3.265 | 93 (89) |
| Lithuania | 0.856484049109405 | 3.187(2.678 to 3.728) | 0.377 | 2.809 | 84 (73) |
| Luxembourg | 0.884428955125693 | 0.428(0.363 to 0.502) | 0.403 | 0.025 | 6 (3) |
| Madagascar | 0.40024694263784 | 6.651(4.48 to 9.591) | 1.903 | 4.748 | 121 (128) |
| Malawi | 0.384553633730301 | 8.881(6.247 to 12.368) | 1.902 | 6.98 | 152 (157) |
| Malaysia | 0.742523828414751 | 4.084(3.158 to 5.143) | 0.687 | 3.397 | 97 (90) |
| Maldives | 0.650886627343107 | 2.614(1.805 to 3.627) | 0.738 | 1.875 | 65 (64) |
| Mali | 0.268579940925684 | 6.333(4.433 to 8.926) | 2.586 | 3.747 | 104 (124) |
| Malta | 0.801585033998971 | 1.448(1.251 to 1.676) | 0.472 | 0.976 | 38 (35) |
| Marshall Islands | 0.574091128177309 | 44.311(26.462 to 69.657) | 0.749 | 43.562 | 202 (202) |
| Mauritania | 0.498945100442996 | 4.619(3.054 to 6.829) | 1.461 | 3.158 | 89 (97) |
| Mauritius | 0.718260445552653 | 24.944(21.999 to 27.801) | 0.683 | 24.261 | 197 (197) |
| Mexico | 0.664575304259183 | 14.677(12.96 to 16.612) | 0.734 | 13.943 | 182 (182) |
| Micronesia (Federated States of) | 0.587534967103163 | 29.243(19.659 to 42.824) | 0.763 | 28.48 | 199 (199) |
| Monaco | 0.908262830989574 | 0.568(0.348 to 0.864) | 0.373 | 0.195 | 15 (9) |
| Mongolia | 0.617621564638127 | 2.78(2.007 to 3.671) | 0.743 | 2.037 | 70 (65) |
| Montenegro | 0.795800584021403 | 2.471(1.855 to 3.198) | 0.461 | 2.01 | 69 (61) |
| Morocco | 0.562698300927195 | 2.991(1.889 to 4.571) | 0.757 | 2.234 | 74 (70) |
| Mozambique | 0.326462613636377 | 11.95(7.61 to 17.501) | 1.916 | 10.034 | 172 (176) |
| Myanmar | 0.533900839611906 | 12.106(8.454 to 16.842) | 1.453 | 10.653 | 175 (177) |
| Namibia | 0.617564872149664 | 6.248(3.815 to 9.383) | 0.748 | 5.5 | 136 (123) |
| Nauru | 0.625177833565553 | 39.856(26.702 to 57.319) | 0.745 | 39.111 | 201 (201) |
| Nepal | 0.43317463481343 | 4.544(3.052 to 6.677) | 1.905 | 2.64 | 81 (96) |
| Netherlands | 0.888464256397732 | 0.751(0.669 to 0.837) | 0.362 | 0.389 | 20 (16) |
| New Zealand | 0.849442498970719 | 1.369(1.241 to 1.499) | 0.46 | 0.909 | 36 (32) |
| Nicaragua | 0.523958472235925 | 5.804(4.304 to 7.677) | 1.432 | 4.371 | 116 (115) |
| Niger | 0.168072773803995 | 3.794(2.443 to 5.746) | 3.701 | 0.094 | 7 (85) |
| Nigeria | 0.503390832763244 | 5.127(3.524 to 7.075) | 1.452 | 3.675 | 101 (106) |
| Niue | 0.726222049709539 | 23.182(15.198 to 34.777) | 0.681 | 22.502 | 195 (195) |
| North Macedonia | 0.750629703270047 | 2.787(2.008 to 3.737) | 0.656 | 2.131 | 71 (66) |
| Northern Mariana Islands | 0.771535213383281 | 11.262(8.376 to 14.853) | 0.466 | 10.796 | 176 (174) |
| Norway | 0.916132810119013 | 0.975(0.925 to 1.027) | 0.378 | 0.597 | 25 (20) |
| Oman | 0.773391601871737 | 4.195(2.941 to 5.756) | 0.506 | 3.689 | 102 (93) |
| Pakistan | 0.504028688613448 | 7.299(5.37 to 9.813) | 1.456 | 5.843 | 140 (141) |
| Palau | 0.75404693141209 | 24.411(16.85 to 34.233) | 0.603 | 23.808 | 196 (196) |
| Palestine | 0.631011665054344 | 3.926(2.907 to 5.165) | 0.76 | 3.165 | 90 (88) |
| Panama | 0.708864827518009 | 4.753(3.687 to 5.917) | 0.708 | 4.045 | 109 (101) |
| Papua New Guinea | 0.417797443084353 | 21.884(15.578 to 30.053) | 1.898 | 19.986 | 190 (192) |
| Paraguay | 0.635718098852038 | 7.33(5.251 to 9.961) | 0.746 | 6.584 | 150 (143) |
| Peru | 0.662054036755862 | 3.113(2.159 to 4.384) | 0.736 | 2.377 | 76 (71) |
| Philippines | 0.651219328702707 | 9.082(7.611 to 10.685) | 0.75 | 8.332 | 161 (158) |
| Poland | 0.812042808667712 | 2.28(2.06 to 2.497) | 0.457 | 1.824 | 63 (53) |
| Portugal | 0.744151850665712 | 0.993(0.87 to 1.12) | 0.693 | 0.3 | 19 (21) |
| Puerto Rico | 0.825525846914029 | 9.238(7.437 to 11.347) | 0.501 | 8.737 | 163 (159) |
| Qatar | 0.846860584038945 | 3.682(2.495 to 5.272) | 0.459 | 3.224 | 92 (81) |
| Republic of Korea | 0.886675266756997 | 1.406(1.124 to 1.779) | 0.394 | 1.013 | 39 (34) |
| Republic of Moldova | 0.732214875427507 | 3.155(2.729 to 3.659) | 0.692 | 2.464 | 78 (72) |
| Romania | 0.768453864365965 | 1.093(0.921 to 1.277) | 0.499 | 0.594 | 24 (25) |
| Russian Federation | 0.808536004906566 | 2.296(2.101 to 2.479) | 0.475 | 1.822 | 62 (55) |
| Rwanda | 0.435588705789772 | 6.905(3.717 to 11.299) | 1.897 | 5.008 | 127 (135) |
| Saint Kitts and Nevis | 0.754987054945241 | 5.67(4.202 to 7.644) | 0.565 | 5.105 | 129 (111) |
| Saint Lucia | 0.672509735365292 | 10.701(8.54 to 13.24) | 0.738 | 9.963 | 171 (170) |
| Saint Vincent and the Grenadines | 0.637195962512539 | 15.794(13.185 to 18.867) | 0.743 | 15.051 | 184 (184) |
| Samoa | 0.593392769469481 | 20.131(13.475 to 29.31) | 0.742 | 19.389 | 189 (189) |
| San Marino | 0.888005473958106 | 0.402(0.212 to 0.653) | 0.388 | 0.014 | 4 (2) |
| Sao Tome and Principe | 0.505413746708186 | 2.512(1.592 to 3.799) | 1.434 | 1.078 | 41 (62) |
| Saudi Arabia | 0.815143493214829 | 5.982(4.002 to 8.558) | 0.482 | 5.5 | 135 (118) |
| Senegal | 0.408054192634391 | 6.562(4.377 to 9.555) | 1.902 | 4.66 | 120 (126) |
| Serbia | 0.792416293685483 | 2.389(1.796 to 3.076) | 0.491 | 1.898 | 66 (57) |
| Seychelles | 0.730150774629382 | 4.712(3.635 to 5.953) | 0.713 | 3.999 | 108 (100) |
| Sierra Leone | 0.3586658807038 | 5.259(3.5 to 7.769) | 1.902 | 3.357 | 95 (107) |
| Singapore | 0.856097766112729 | 0.331(0.293 to 0.374) | 0.331 | 0 | 1.5 (1) |
| Slovakia | 0.810610529921693 | 1.367(1.024 to 1.772) | 0.472 | 0.895 | 35 (31) |
| Slovenia | 0.842430731281465 | 1.118(0.908 to 1.336) | 0.464 | 0.655 | 28 (26) |
| Solomon Islands | 0.429360316406646 | 39.538(26.857 to 55.938) | 1.895 | 37.643 | 200 (200) |
| Somalia | 0.0776881089723748 | 10.547(6.655 to 15.947) | 10.547 | 0 | 1.5 (167) |
| South Africa | 0.679626598156588 | 8.83(7.637 to 10.044) | 0.746 | 8.084 | 160 (156) |
| South Sudan | 0.278371125484101 | 9.895(6.403 to 14.724) | 1.967 | 7.928 | 157 (163) |
| Spain | 0.769283697979998 | 0.476(0.417 to 0.536) | 0.457 | 0.019 | 5 (4) |
| Sri Lanka | 0.70153493519423 | 6.73(3.986 to 9.91) | 0.734 | 5.996 | 143 (131) |
| Sudan | 0.54194973514933 | 2.909(1.72 to 4.55) | 1.452 | 1.457 | 53 (68) |
| Suriname | 0.63366573910901 | 9.669(6.857 to 13.133) | 0.763 | 8.907 | 166 (162) |
| Sweden | 0.886880298870342 | 1.091(0.936 to 1.255) | 0.38 | 0.711 | 30 (24) |
| Switzerland | 0.933059110986638 | 0.482(0.423 to 0.548) | 0.374 | 0.108 | 8 (5) |
| Syrian Arab Republic | 0.62300407463119 | 2.312(1.549 to 3.414) | 0.741 | 1.571 | 55 (56) |
| Taiwan (Province of China) | 0.874747053187929 | 3.609(3.154 to 4.061) | 0.406 | 3.203 | 91 (80) |
| Tajikistan | 0.541511187051998 | 3.449(2.421 to 4.697) | 1.445 | 2.004 | 68 (78) |
| Thailand | 0.682547932974593 | 6.186(4.287 to 8.508) | 0.739 | 5.447 | 134 (122) |
| Timor-Leste | 0.444667618881833 | 3.694(2.483 to 5.362) | 1.897 | 1.797 | 61 (82) |
| Togo | 0.408533695477986 | 5.825(3.71 to 8.799) | 1.911 | 3.914 | 105 (116) |
| Tokelau | 0.68642562083005 | 18.417(12.97 to 26.319) | 0.735 | 17.682 | 186 (185) |
| Tonga | 0.626349935807987 | 21.435(14.747 to 30.564) | 0.745 | 20.691 | 192 (190) |
| Trinidad and Tobago | 0.768763253980677 | 18.834(13.764 to 24.597) | 0.464 | 18.37 | 188 (188) |
| Tunisia | 0.682432215876962 | 2.036(1.317 to 2.94) | 0.735 | 1.301 | 47 (46) |
| Turkey | 0.712692673098478 | 2.044(1.476 to 2.767) | 0.718 | 1.326 | 49 (48) |
| Turkmenistan | 0.682160775869611 | 6.784(5.133 to 9.044) | 0.733 | 6.052 | 144 (134) |
| Tuvalu | 0.576620529344684 | 22.837(15.76 to 31.705) | 0.774 | 22.063 | 193 (194) |
| Uganda | 0.423261181495743 | 7.659(4.728 to 12.216) | 1.898 | 5.76 | 139 (147) |
| Ukraine | 0.760773912846782 | 1.651(1.146 to 2.203) | 0.562 | 1.089 | 42 (38) |
| United Arab Emirates | 0.849317734035032 | 1.902(1.215 to 2.759) | 0.455 | 1.446 | 52 (43) |
| United Kingdom | 0.859000181772086 | 0.95(0.92 to 0.978) | 0.374 | 0.575 | 23 (19) |
| United Republic of Tanzania | 0.446568273334685 | 6.668(4.377 to 9.557) | 1.897 | 4.771 | 122 (130) |
| United States Virgin Islands | 0.821830853383701 | 7.997(5.205 to 11.351) | 0.463 | 7.534 | 155 (150) |
| United States of America | 0.862448354015145 | 3.774(3.624 to 3.915) | 0.385 | 3.39 | 96 (84) |
| Uruguay | 0.719283444839432 | 1.98(1.749 to 2.219) | 0.701 | 1.279 | 46 (45) |
| Uzbekistan | 0.662621694181816 | 4.903(3.913 to 5.956) | 0.737 | 4.166 | 112 (105) |
| Vanuatu | 0.473100705820327 | 21.769(14.827 to 31.765) | 1.641 | 20.128 | 191 (191) |
| Venezuela (Bolivarian Republic of) | 0.596513058857335 | 7.064(5.204 to 9.419) | 0.754 | 6.31 | 146 (139) |
| Viet Nam | 0.627933720680671 | 4.665(3.209 to 6.6) | 0.743 | 3.922 | 106 (98) |
| Yemen | 0.450376375244811 | 2.036(1.192 to 3.545) | 1.901 | 0.135 | 12 (47) |
| Zambia | 0.505948954434909 | 9.382(6.07 to 13.86) | 1.465 | 7.918 | 156 (161) |
| Zimbabwe | 0.473819486202707 | 6.658(4.32 to 9.77) | 1.754 | 4.904 | 125 (129) |

**SDI: Socio-Demographic Index.**
